# Identification of differential proteomics in Epstein-Barr virus-associated gastric cancer and related functional analysis

**DOI:** 10.1186/s12935-021-02077-6

**Published:** 2021-07-12

**Authors:** Zeyang Wang, Zhi Lv, Qian Xu, Liping Sun, Yuan Yuan

**Affiliations:** 1 Tumor Etiology and Screening Department of Cancer Institute and General Surgery, The First Hospital of China Medical University, No.155 NanjingBei Street, Heping District, Shenyang, 110001 Liaoning Province China; 2 Key Laboratory of Cancer Etiology and Prevention in Liaoning Education Department, The First Hospital of China Medical University, Shenyang, 110001 China; 3 Key Laboratory of GI Cancer Etiology and Prevention in Liaoning Province, The First Hospital of China Medical University, Shenyang, 110001 China

**Keywords:** EBV, Gastric cancer, Proteomics, Function, GBP5

## Abstract

**Background:**

Epstein-Barr virus-associated gastric cancer (EBVaGC) is the most common EBV-related malignancy. A comprehensive research for the protein expression patterns in EBVaGC established by high-throughput assay remains lacking. In the present study, the protein profile in EBVaGC tissue was explored and related functional analysis was performed.

**Methods:**

Epstein-Barr virus-encoded RNA (EBER) in situ hybridization (ISH) was applied to EBV detection in GC cases. Data-independent acquisition (DIA) mass spectrometry (MS) was performed for proteomics assay of EBVaGC. Functional analysis of identified proteins was conducted with bioinformatics methods. Immunohistochemistry (IHC) staining was employed to detect protein expression in tissue.

**Results:**

The proteomics study for EBVaGC was conducted with 7 pairs of GC cases. A total of 137 differentially expressed proteins in EBV-positive GC group were identified compared with EBV-negative GC group. A PPI network was constructed for all of them, and several proteins with relatively high interaction degrees could be the hub genes in EBVaGC. Gene enrichment analysis showed they might be involved in the biological pathways related to energy and biochemical metabolism. Combined with GEO datasets, a highly associated protein (GBP5) with EBVaGC was screened out and validated with IHC staining. Further analyses demonstrated that GBP5 protein might be associated with clinicopathological parameters and EBV infection in GC.

**Conclusions:**

The newly identified proteins with significant differences and potential central roles could be applied as diagnostic markers of EBVaGC. Our study would provide research clues for EBVaGC pathogenesis as well as novel targets for the molecular-targeted therapy of EBVaGC.

**Supplementary Information:**

The online version contains supplementary material available at 10.1186/s12935-021-02077-6.

## Background

Epstein-Barr virus (EBV) is a ubiquitous human herpes virus originally discovered in Burkitt lymphoma [1]. It has been recognized as the primary virus to be directly involved in numerous malignant tumors. EBV-associated gastric cancer (EBVaGC) is the most common one among EBV-related malignancies. And it accounts for nearly 10% of gastric carcinoma worldwide with variable frequencies between geographic regions [2]. EBVaGC was also identified as one of the four molecular subtypes of GC according to a full-scale molecular genetic analysis published by the Cancer Genome Atlas (TCGA) [3]. The diverse properties of EBVaGC distinct from other GC types have been attracting extensive attention in the past thirty years, including unique epidemiological, pathological, clinical and molecular features.

The molecular patterns in EBVaGC are complicated comprised of various genetic and epigenetic abnormalities [4]. In any event, cellular gene expression plays a critical role in viral oncogenesis, thus it is quite necessary to clarify the differential proteins with their specific effects on EBVaGC. The proteomics research for infection of pathogenic microorganisms has been rapidly developing since proposed [5, 6]. It aims to figure out the key proteins that determine crucial biological activities encompassing pathogen infection and host defense, and also the mechanisms for these proteins to function. Great significance has been manifested in the proteomics of both pathogens in vitro or vivo and infected tissue or cells of host, especially for some common organisms such as Salmonella typhimurium, Shigella flexneri and Helicobacter pylori, etc. [7–9]. The identification of proteomic differences for important organisms may not only conduce to in-depth knowledge of their pathogenesis, but also provide novel targets for the treatment of related diseases [10]. As for EBV infection-induced GC, however, almost all current studies at protein level were focused on single element or large-scale datasets based on bioinformatics database [11]. A comprehensive research for the protein expression patterns in EBVaGC established by high-throughput assay remains lacking.

In the present study, the protein profile in EBVaGC tissue was explored and differentially expressed proteins between EBV-positive and negative GC was identified. Functional analysis was subsequently performed for the differential proteins. Furthermore, validation experiment and related analyses were conducted for highly associated protein. We intend to make a deeper illustration for the molecular patterns involved in EBVaGC pathogenesis, as well as provide new clues for the molecular-targeted therapy of EBVaGC.

## Methods

### Sample preparation

The ethics committee of the First Hospital of China Medical University has approved the project. Signed informed consents were obtained from every participant. The subjects enrolled in this study were GC patients receiving surgical treatment in our hospital from September 2012 to October 2019. Screening criteria were having no other primary tumors and not undergoing any preoperative radiochemotherapy. Gastric tissue specimens were gained from each patient after surgical operation including cancer with adjacent cancer-free tissue. Two senior gastrointestinal pathologists made the histopathological diagnosis independently. Fresh GC tissue and adjacent normal tissue were randomly taken out from each case and divided into several parts with the size to fit for single use. Samples for EBV detection, hematoxylin–eosin (HE) staining and immunohistochemical staining were fixed with 10% formalin and embedded in paraffin. And samples for proteomics research were frozen in liquid nitrogen immediately and stored at − 80 °C.

### Determination of EBV infection in GC

Epstein-Barr virus-encoded RNA (EBER) in situ hybridization (ISH) was applied to EBV detection for 140 GC cases using an EBER test kit (Beijing Zhongshan Jinqiao). In brief, tissue paraffin sections were cut into 4–6 μm-thick pretreated with dimethylbenzene and 100% ethanol. Each slice was incubated with 300–400 μl gastric enzyme for 30 min at 37℃. After dehydration by gradient ethanol, we added 10–20 μl EBER probe solution on each slice for hybridization and incubated them in moist chamber for 1 h at 37 °C. Then the sections were washed with PBS and incubated with peroxidase-labeled anti-digoxin antibody for 30 min at 37 °C. Finally, all tissue sections were stained with DAB (5-15 min) and restained with hematoxylin (5–10 s).

### Quantitative proteomics of EBVaGC

Data-independent acquisition (DIA) mass spectrometry (MS) was performed by Genechem Co., Ltd. (Shanghai, China) to assay the proteomics of EBVaGC [12]. Briefly, total protein was extracted from tissue specimens and measured with BCA kit. We took 20 μg protein from each extract and mixed them with 6X sample loading buffer. The solutions were tested by SDS-PAGE (250 V, 40 min) and the gels were stained with Coomassie Blue. Filter-aided sample preparation (FASP) was adopted to extract and quantify peptides from 200 μg protein solution. All the peptides mix were graded by 1260 infinity II high performance liquid chromatography (HPLC) system (Agilent Technologies Inc.). We collected 48 components and 12 fractions after merging. 6 μl sample was taken from each fraction, mixed with 1 μl 10 × iRT peptides and separated by nano-LC. Finally, DIA-based MS analysis was conducted with LC–MS including Easy nLC system (Thermo Fisher Scientific) and Oribitrap Fusion Lumos system (Thermo Fisher Scientific). In addition, the MS based on data-dependent acquisition (DDA) was also performed and a spectrogram database was established for quality control.

### Determination of protein expression in tissue

Immunohistochemistry (IHC) staining was employed to detect protein expression in tissue [13]. In short, paraffin-embedded tissue specimens were cut into 4 μm-thick sections. Tissue sections were dewaxed, rehydrated with gradient ethanol, incubated in 10 mmol/l citrate buffer (pH 6.0) and heated for 90 s. Endogenous peroxidase was blocked with 3% hydrogen peroxide (10 min). Tissue collagen was spoilt with 10% normal goat serum (10 min) for reducing non-specific binding. Rabbit polyclonal antibody for target protein (Abcam, UK) was used as primary antibody to incubate the samples for 1 h at room temperature. After washing with PBS, the samples were incubated with biotin-labeled secondary antibody (Fuzhou Maixin Biotech) and followed by streptavidin–horseradish peroxidase (HRP), both for 10 min at room temperature. Then the samples were stained with DAB (DAB-0031, Fuzhou Maixin Biotech), dehydrated and fixed with resin. Finally, the stained tissue sections were observed by experienced pathologists under inverted microscope. IHC staining was scored for each tissue section with positive staining based on the area (25%, 50%, 75%, 100%) and intensity (+ , +  + , +  + +). The final score was set to range from 1 to 4 after conversion.

### Data analysis

The raw data of DIA-MS was processed with Spectronaut Pulsar X (v12, Biognosys AG). After normalization, differentially expressed proteins between EBV-positive and negative GC were identified. The threshold were set as absolute fold change (FC) > 1.5 and *P* < 0.05 corrected with 1% false discovery rate (FDR). Protein–protein interaction (PPI) information was downloaded from the STRING online tool (v11.0, https://string-db.org) and PPI network was constructed with Cytoscape software (v3.6.1). Funrich database (v3.1.3) was applied to gene enrichment analysis including expression site, Gene Ontology (GO) and biological pathways. The online datasets of gene expression profiling by microarray about EBVaGC were searched in Gene Expression Omnibus (GEO) database and analyzed with GEO2R package. Data processing and mapping was performed using R-project (v4.0.3) and Rstudio software (v1.3.1093). SPSS (v22.0) software was employed to analyze the data of validation experiments, including *χ*^2^ test, independent *t* test or Mann–Whitney *U* test, Kaplan–Meier test, log rank test and Cox regression, etc.. All the tests were judged as statistically significant when |FC| > 2.0 and *P* < 0.05 after correction with Benjamini-Hochberg (BH) method (FDR).

## Results

### Identification of EBVaGC subjects

Based on the proven method of EBER-ISH, the nucleus of EBV-infected cells could be strongly stained after disposal following kit instructions [14]. A total of 7 tissue specimens with positive EBER signals out of the 140 GC cases were identified as EBV-positive GC group (A1-A7, Additional file 2: Fig. S1). Meanwhile, another 7 GC samples without positive staining were picked as EBV-negative GC group (B1–B7) matched by gender and age (± 5 years). The basic information and pathological characteristics of all subjects in the two groups were shown in Additional file 1: Table S1.

### Characteristics of the protein profile in EBVaGC

**Fig. 1:**
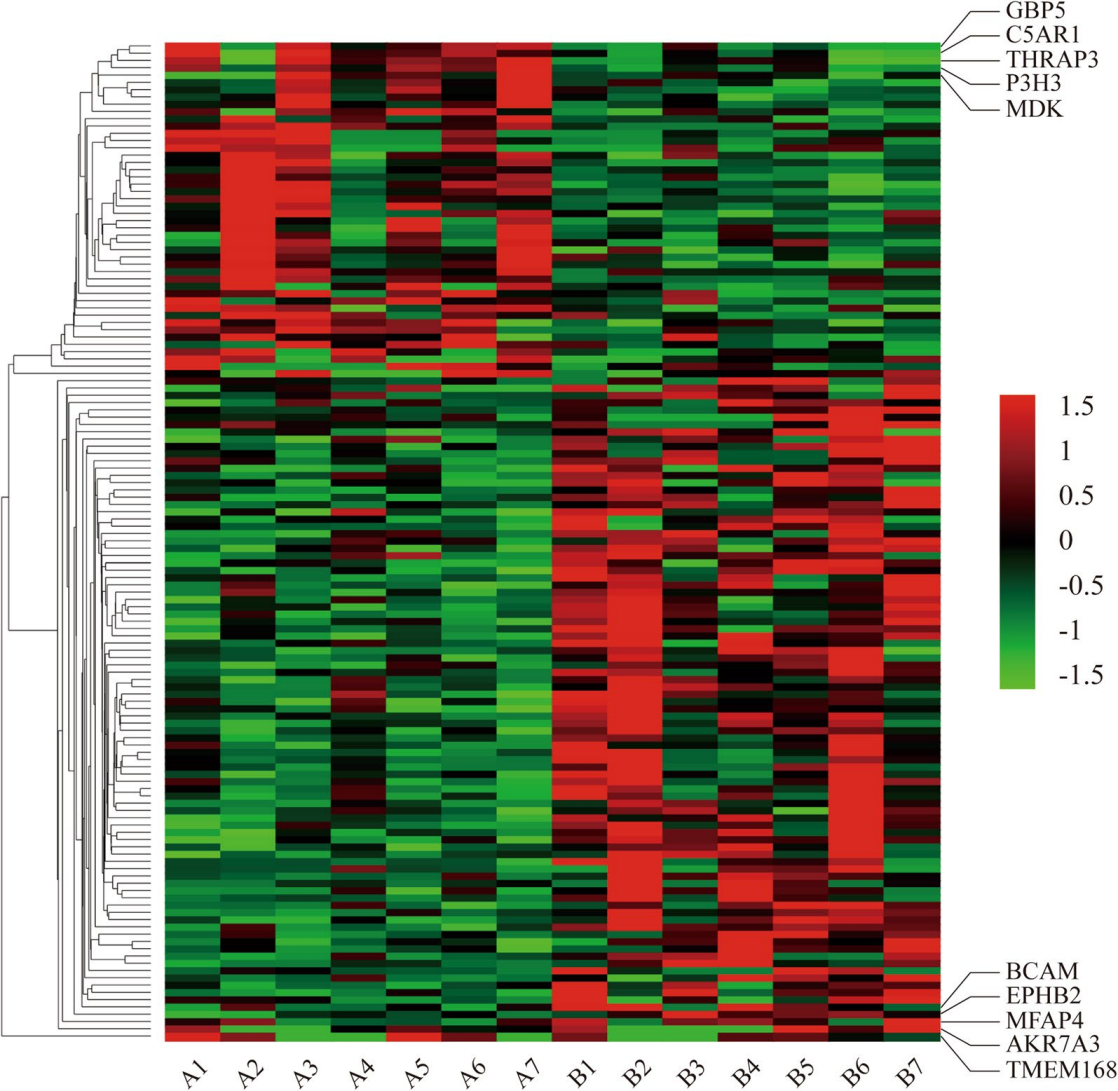
The clustered heat map of the differentially expressed proteins in EBVaGC. Several representative proteins are labeled

The proteomics study for EBVaGC was conducted with the above 7 pairs of GC cases. A total of 137 differentially expressed proteins in EBV-positive GC group were identified compared with EBV-negative GC group (Table 1). Among them, GBP5, C5AR1, THRAP3, P3H3 and MDK were the top 5 differential proteins in the 47 up-regulated records. For the 90 down-regulated proteins, TMEM168, AKR7A3, MFAP4, EPHB2 and BCAM had the top 5 FC values. The clustered expression profile of all differential proteins in assayed tissue was shown in Fig. 1. And their detailed expression levels in each sample were listed in Additional file 1: Table S2.

**Table 1:** The differentially expressed proteins between EBV-positive and negative GC

Genes	Protein description	FC (abs)	*P* value	Regulation
GBP5	Guanylate-binding protein 5	3.45	0.028	Up
C5AR1	C5a anaphylatoxin chemotactic receptor 1	3.39	0.038	Up
THRAP3	Thyroid hormone receptor-associated protein 3	3.25	0.002	Up
P3H3	Prolyl 3-hydroxylase 3	3.10	0.035	Up
MDK	Midkine	3.07	0.042	Up
ALOX5AP	Arachidonate 5-lipoxygenase-activating protein	2.84	0.048	Up
BPI	Bactericidal permeability-increasing protein	2.69	0.025	Up
HLA-DRB1	HLA class II histocompatibility antigen, DRB1-12 beta chain	2.56	0.015	Up
PPL	Periplakin	2.49	0.027	Up
ISLR	Immunoglobulin superfamily containing leucine-rich repeat protein	2.31	0.047	Up
APOL2	Apolipoprotein L2	2.29	0.009	Up
HCK	Tyrosine-protein kinase HCK	2.21	0.020	Up
AKAP2	A-kinase anchor protein 2	2.17	0.026	Up
ITGA11	Integrin alpha-11	2.14	0.024	Up
ITGB2	Integrin beta-2	2.13	0.025	Up
COQ6	Ubiquinone biosynthesis monooxygenase COQ6, mitochondrial	2.09	0.039	Up
DENND1C	DENN domain-containing protein 1C	2.06	0.002	Up
RAB31	Ras-related protein Rab-31	2.05	0.041	Up
CYBA	Cytochrome b-245 light chain	2.02	0.001	Up
FCGR3A	Low affinity immunoglobulin gamma Fc region receptor III-A	1.94	0.044	Up
CYBB	Cytochrome b-245 heavy chain	1.90	0.007	Up
KEAP1	Kelch-like ECH-associated protein 1	1.88	0.003	Up
KALRN	Kalirin	1.86	< 0.001	Up
GBP1	Guanylate-binding protein 1	1.85	0.020	Up
DPYD	Dihydropyrimidine dehydrogenase [NADP( +)]	1.81	0.049	Up
TOR1B	Torsin-1B	1.80	0.014	Up
CNN2	Calponin-2	1.78	0.041	Up
TCIRG1	V-type proton ATPase 116 kDa subunit a isoform 3	1.78	0.023	Up
TAP1	Antigen peptide transporter 1	1.76	0.037	Up
SRRM2	Serine/arginine repetitive matrix protein 2	1.75	0.026	Up
CD40	Tumor necrosis factor receptor superfamily member 5	1.74	0.036	Up
FUT8	Alpha-(1,6)-fucosyltransferase	1.71	0.037	Up
SCAF1	Splicing factor, arginine/serine-rich 19	1.69	0.044	Up
TLR3	Toll-like receptor 3	1.66	0.020	Up
GRN	Granulins	1.65	0.029	Up
NSA2	Ribosome biogenesis protein NSA2 homolog	1.64	0.050	Up
CLASP1	CLIP-associating protein 1	1.61	0.033	Up
CPOX	Oxygen-dependent coproporphyrinogen-III oxidase, mitochondrial	1.61	0.023	Up
ATP6AP1	V-type proton ATPase subunit S1	1.60	0.022	Up
CARHSP1	Calcium-regulated heat-stable protein 1	1.60	0.037	Up
LPCAT2	Lysophosphatidylcholine acyltransferase 2	1.59	0.040	Up
GALNT2	Polypeptide N-acetylgalactosaminyltransferase 2	1.59	0.038	Up
COMMD10	COMM domain-containing protein 10	1.59	0.032	Up
ATP6V1D	V-type proton ATPase subunit D	1.57	0.020	Up
LRRC40	Leucine-rich repeat-containing protein 40	1.54	0.011	Up
PREX1	Phosphatidylinositol 3,4,5-trisphosphate-dependent Rac exchanger 1 protein	1.53	0.029	Up
GBP2	Guanylate-binding protein 2	1.53	0.023	Up
PEBP1	Phosphatidylethanolamine-binding protein 1	1.51	0.016	Down
UBR5	E3 ubiquitin-protein ligase UBR5	1.51	0.049	Down
TXN2	Thioredoxin, mitochondrial	1.52	0.011	Down
ADD1	Alpha-adducin	1.52	0.019	Down
EPB41L1	Band 4.1-like protein 1	1.52	0.033	Down
IDI1	Isopentenyl-diphosphate Delta-isomerase 1	1.54	0.009	Down
EML2	Echinoderm microtubule-associated protein-like 2	1.55	0.035	Down
ATP1B1	Sodium/potassium-transporting ATPase subunit beta-1	1.55	0.035	Down
EIF4A2	Eukaryotic initiation factor 4A-II	1.56	0.004	Down
MRI1	Methylthioribose-1-phosphate isomerase	1.56	0.009	Down
CST3	Cystatin-C	1.56	0.035	Down
ABHD14B	Protein ABHD14B	1.57	0.013	Down
ARFIP2	Arfaptin-2	1.58	0.021	Down
ATPAF2	ATP synthase mitochondrial F1 complex assembly factor 2	1.58	0.014	Down
PSMG4	Proteasome assembly chaperone 4	1.59	0.036	Down
ECSIT	Evolutionarily conserved signaling intermediate in Toll pathway, mitochondrial	1.59	0.030	Down
RNMT	mRNA cap guanine-N7 methyltransferase	1.59	0.019	Down
CD46	Membrane cofactor protein	1.61	0.022	Down
SUPV3L1	ATP-dependent RNA helicase SUPV3L1, mitochondrial	1.61	0.042	Down
DTD1	D-aminoacyl-tRNA deacylase 1	1.61	0.009	Down
FAM213A	Redox-regulatory protein FAM213A	1.63	0.019	Down
C11orf54	Ester hydrolase C11orf54	1.63	0.049	Down
BCKDHB	2-oxoisovalerate dehydrogenase subunit beta, mitochondrial	1.64	0.012	Down
GFPT1	Glutamine–fructose-6-phosphate aminotransferase [isomerizing] 1	1.64	0.043	Down
EPB41L2	Band 4.1-like protein 2	1.64	0.035	Down
RAB6D/RAB6C	Ras-related protein Rab-6D/Ras-related protein Rab-6C	1.66	0.025	Down
DAG1	Dystroglycan	1.66	0.018	Down
HEBP2	Heme-binding protein 2	1.67	0.039	Down
QDPR	Dihydropteridine reductase	1.68	0.047	Down
UBE4B	Ubiquitin conjugation factor E4 B	1.68	0.045	Down
NAXE	NAD(P)H-hydrate epimerase	1.68	0.007	Down
GLRX5	Glutaredoxin-related protein 5, mitochondrial	1.70	0.006	Down
PPOX	Protoporphyrinogen oxidase	1.70	0.012	Down
CHRAC1	Chromatin accessibility complex protein 1	1.71	0.048	Down
MPST	3-mercaptopyruvate sulfurtransferase	1.73	0.015	Down
COQ3	Ubiquinone biosynthesis O-methyltransferase, mitochondrial	1.73	0.015	Down
F13A1	Coagulation factor XIII A chain	1.74	0.033	Down
SGCD	Delta-sarcoglycan	1.75	0.020	Down
NFU1	NFU1 iron-sulfur cluster scaffold homolog, mitochondrial	1.75	0.016	Down
TXLNG	Gamma-taxilin	1.76	0.010	Down
NRM	Nurim	1.78	0.026	Down
ACAA2	3-ketoacyl-CoA thiolase, mitochondrial	1.78	0.015	Down
TXNL4A	Thioredoxin-like protein 4A	1.80	0.024	Down
F11R	Junctional adhesion molecule A	1.80	0.009	Down
H2AFY2	Core histone macro-H2A.2	1.81	0.020	Down
SPRYD4	SPRY domain-containing protein 4	1.82	0.049	Down
RIDA	2-iminobutanoate/2-iminopropanoate deaminase	1.83	0.012	Down
MLYCD	Malonyl-CoA decarboxylase, mitochondrial	1.85	0.007	Down
ACY1	Aminoacylase-1	1.87	0.001	Down
CDC5L	Cell division cycle 5-like protein	1.88	0.018	Down
ACSS2	Acetyl-coenzyme A synthetase, cytoplasmic	1.89	0.014	Down
DARS2	Aspartate–tRNA ligase, mitochondrial	1.94	0.014	Down
2-Mar	Mitochondrial amidoxime reducing component 2	1.96	0.008	Down
CA1	Carbonic anhydrase 1	1.99	0.025	Down
BRK1	Protein BRICK1	2.00	0.005	Down
CAVIN2	Caveolae-associated protein 2	2.02	0.029	Down
SELENBP1	Methanethiol oxidase	2.03	0.037	Down
COQ8A	Atypical kinase COQ8A, mitochondrial	2.04	0.030	Down
HBG1	Hemoglobin subunit gamma-1	2.07	0.021	Down
PFN2	Profilin-2	2.07	< 0.001	Down
ARHGEF10	Rho guanine nucleotide exchange factor 10	2.08	0.003	Down
GRIP2	Glutamate receptor-interacting protein 2	2.12	0.023	Down
SH3BGRL2	SH3 domain-binding glutamic acid-rich-like protein 2	2.14	0.034	Down
TMEM63A	CSC1-like protein 1	2.18	0.048	Down
CRAT	Carnitine O-acetyltransferase	2.18	0.003	Down
HBE1	Hemoglobin subunit epsilon	2.26	0.036	Down
IGKV2-24	Immunoglobulin kappa variable 2–24	2.28	0.023	Down
VWA5A	von Willebrand factor A domain-containing protein 5A	2.36	0.012	Down
MAOB	Amine oxidase [flavin-containing] B	2.37	0.009	Down
DEPTOR	DEP domain-containing mTOR-interacting protein	2.39	0.013	Down
LTBP4	Latent-transforming growth factor beta-binding protein 4	2.40	0.029	Down
THADA	Thyroid adenoma-associated protein	2.45	0.049	Down
ACSS1	Acetyl-coenzyme A synthetase 2-like, mitochondrial	2.45	0.023	Down
ASS1	Argininosuccinate synthase	2.47	0.013	Down
EPHB3	Ephrin type-B receptor 3	2.54	0.015	Down
ADH1B	Alcohol dehydrogenase 1B	2.64	0.044	Down
HMGCS1	Hydroxymethylglutaryl-CoA synthase, cytoplasmic	2.65	0.046	Down
SLC12A2	Solute carrier family 12 member 2	2.72	0.002	Down
PTGR1	Prostaglandin reductase 1	2.73	0.002	Down
PHGDH	D-3-phosphoglycerate dehydrogenase	2.75	0.005	Down
LRRC1	Leucine-rich repeat-containing protein 1	2.75	0.011	Down
FAF1	FAS-associated factor 1	2.86	0.018	Down
OPLAH	5-oxoprolinase	2.87	0.003	Down
CKMT1A	Creatine kinase U-type, mitochondrial	2.91	0.048	Down
CEP250	Centrosome-associated protein CEP250	3.19	0.004	Down
BCAM	Basal cell adhesion molecule	3.55	0.029	Down
EPHB2	Ephrin type-B receptor 2	3.80	0.047	Down
MFAP4	Microfibril-associated glycoprotein 4	4.09	0.034	Down
AKR7A3	Aflatoxin B1 aldehyde reductase member 3	4.11	0.034	Down
TMEM168	Transmembrane protein 168	4.56	0.011	Down

*EBV* Epstein-Barr virus, *GC* gastric cancer, *FC* (*abs*) absolute fold change

### PPI network of the differentially expressed proteins in EBVaGC

**Fig. 2:**
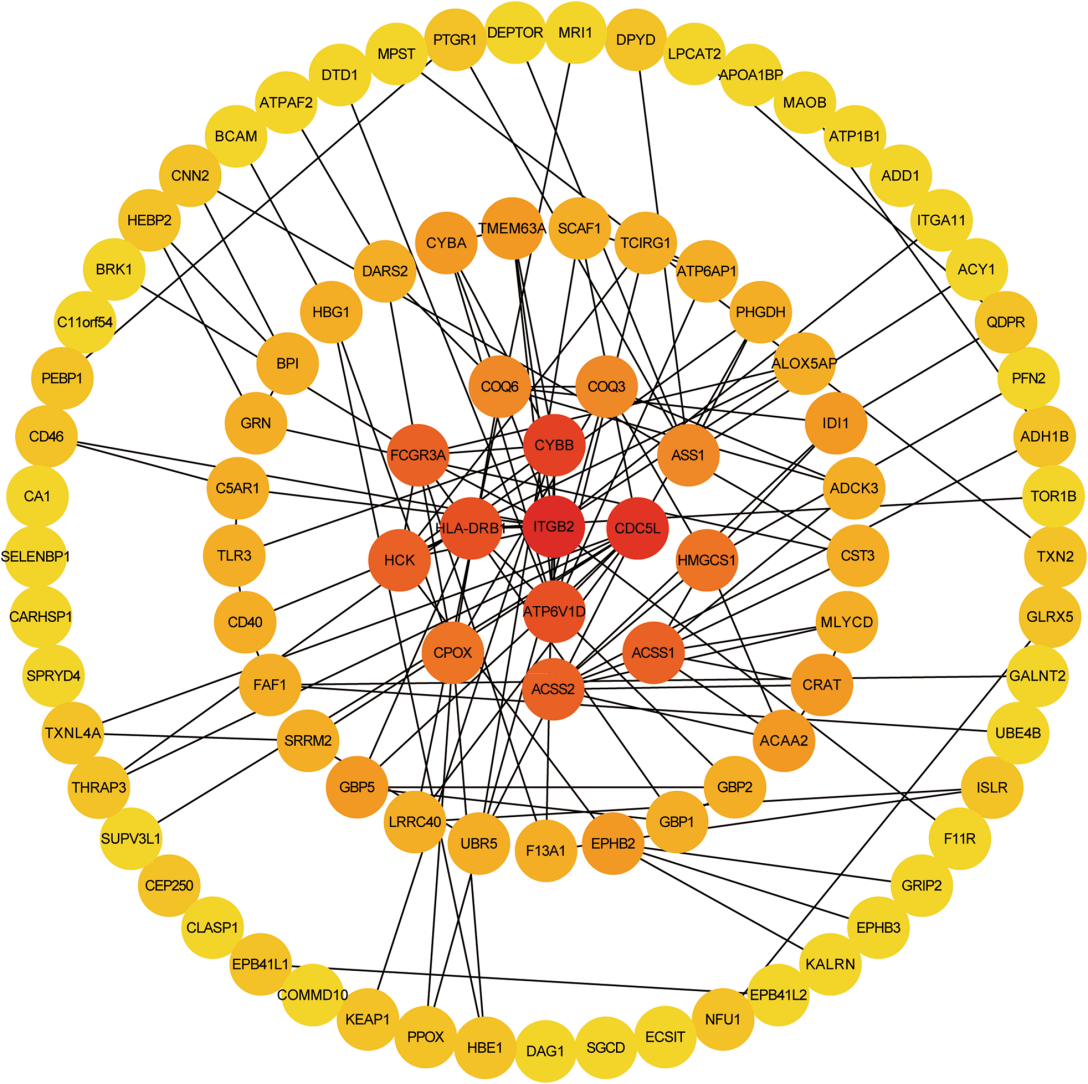
The PPI network of the differentially expressed proteins in EBVaGC. The gradient color of circles from yellow to red represents the interaction degree of proteins from low to high

To investigate the potential gene–gene interactions in EBVaGC, a PPI network was constructed for all above differentially expressed proteins. First, PPI information was collected from the String online database and 96 proteins showed interactions with at least one or more proteins. Based on their interactions and combined scores, the interaction degree for each protein was calculated with the cytoHubba plug-in in Cytoscape software. All the proteins were divided into 5 levels according to their interaction degrees: (1) > 20: 1; (2) 15–20: 4; (3) 10–15: 9; (4) 5–10: 29; and (5) < 5: 53. It was shown that several proteins had relatively high interaction degrees and might be the hub genes in EBVaGC, including ITGB2, CDC5L, CYBB, HLA-DRB1 and ATP6V1D (Fig. 2).

### Gene enrichment analysis of the differentially expressed proteins in EBVaGC

**Fig. 3:**
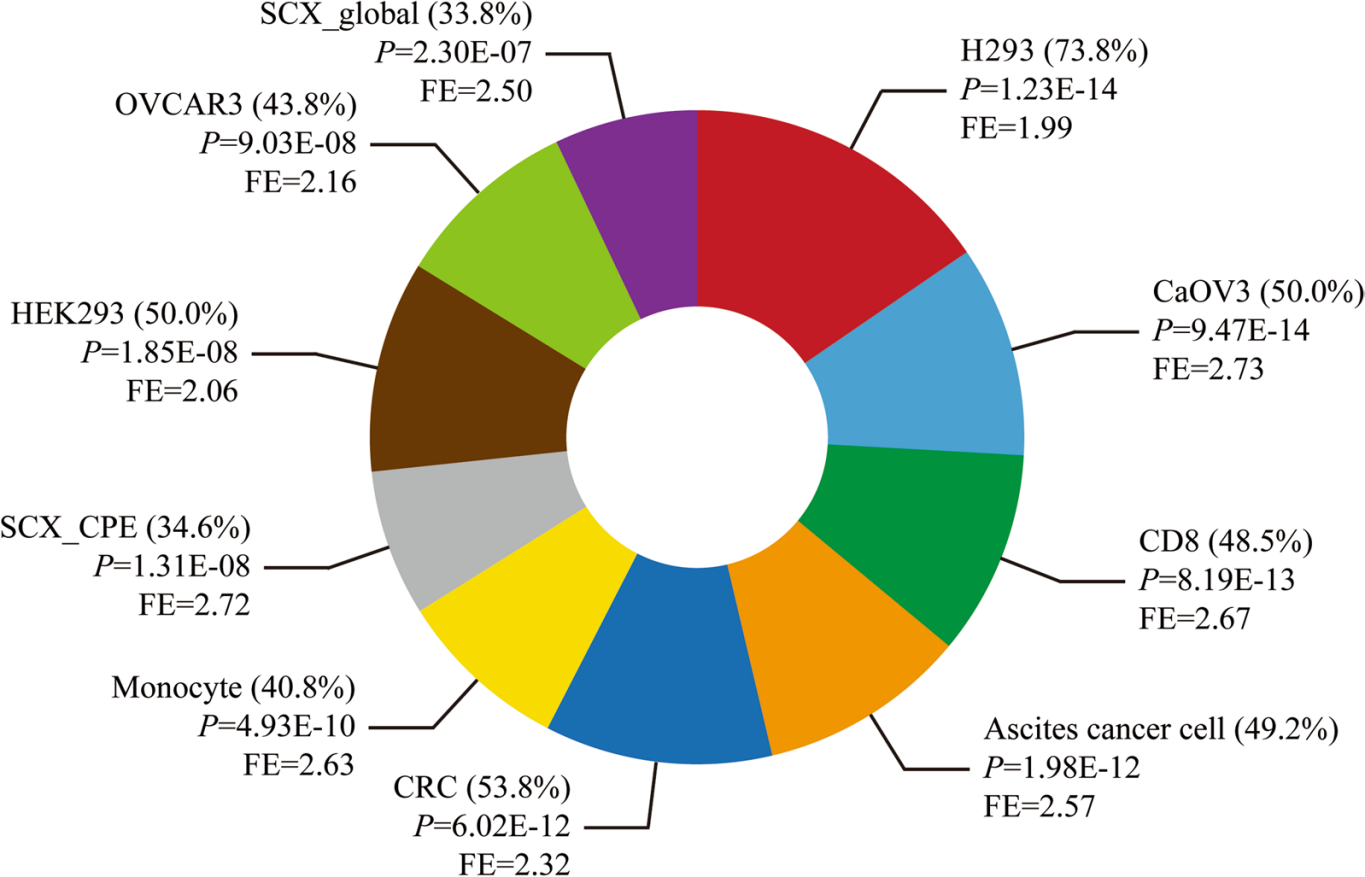
The top 10 significant items in the enrichment analysis of expression sites for the differentially expressed proteins in EBVaGC. FE, fold enrichment

Next, gene enrichment analysis was performed for these differentially expressed proteins to explore their potential biological function involved in EBVaGC. The expression sites of genes were predicted at first, which comprised of diverse cancer tissue, normal tissue and cell lines. The differential proteins between EBV-positive and negative GC were found to be significantly expressed in numerous cell lines and tissue such as H293 cell (*P* = 1.23E-14), CaOV3 cell (*P* = 9.47E-14), CD8 cell (*P* = 8.19E-13), ascites cancer cell (*P* = 1.98E-12) and colorectal cancer (CRC) tissue (*P* = 6.02E-12). Their fold enrichment were 1.99, 2.73, 2.67, 2.57 and 2.32, respectively (Fig. 3).

**Fig. 4:**
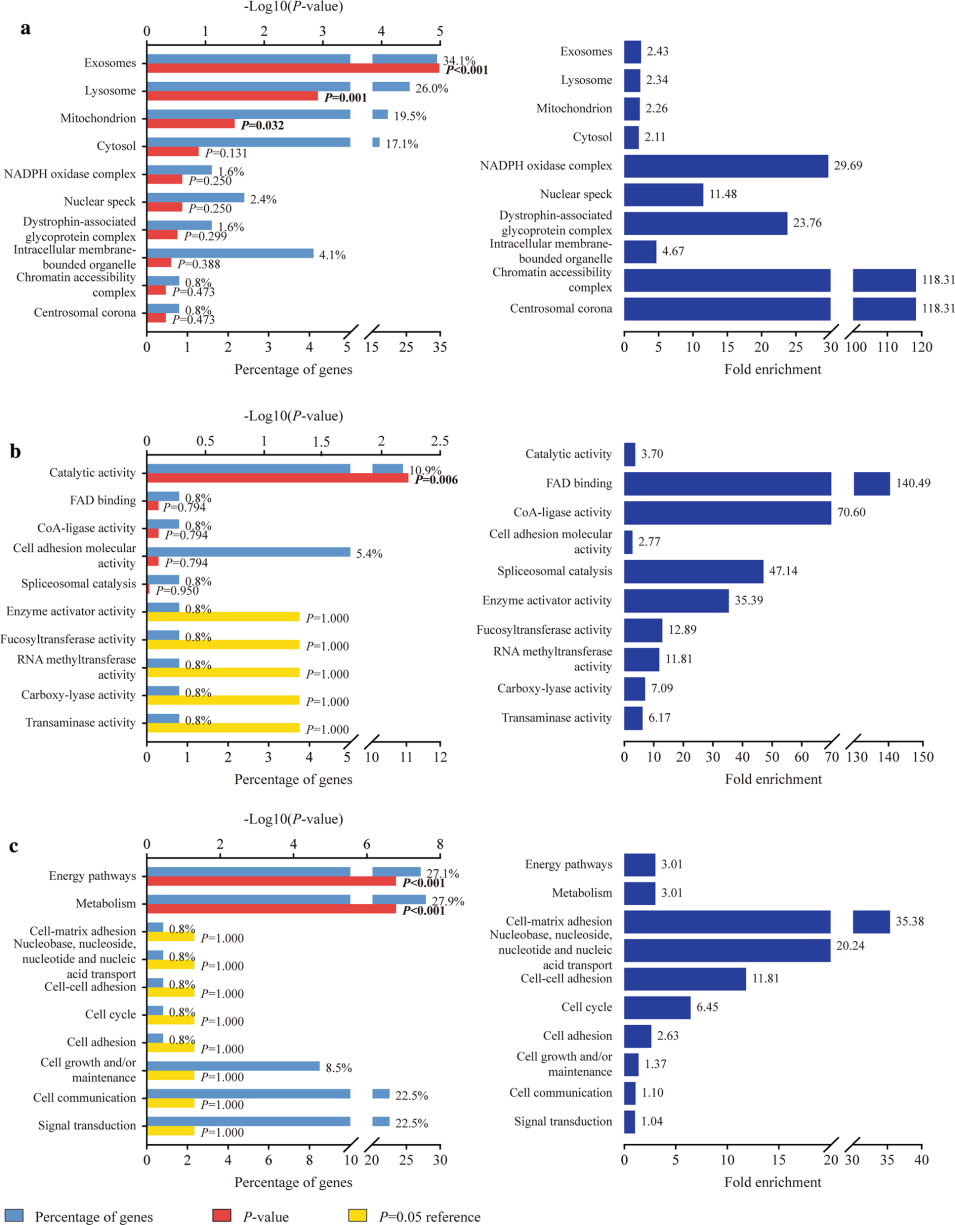
The top 10 significant items in the enrichment analysis of GO-term for the differentially expressed proteins in EBVaGC. **A** cellular component; **B** molecular function; **C** biological process

Then we focused on the GO-term enrichment analysis including cellular component (CC), molecular function (MF) and biological process (BP). Top 10 records sequenced by *P* values were picked for each term. Regarding CC, three items were suggested to significantly enrich the differentially expressed proteins, which were exosomes (*P* < 0.001), lysosome (*P* = 0.001) and mitochondrion (*P* = 0.032). And their fold enrichment respectively were 2.43, 2.34 and 2.26 (Fig. 4A). One term in MF, catalytic activity, showed significant enrichment effect for those proteins (*P* = 0.006, fold enrichment = 3.70, Fig. 4B). As for BP, the differential proteins were observed to be significantly enriched in two items, energy pathways (*P* < 0.001) and metabolism (*P* < 0.001). Both their fold enrichment were 3.01 (Fig. 4C).

**Fig. 5:**
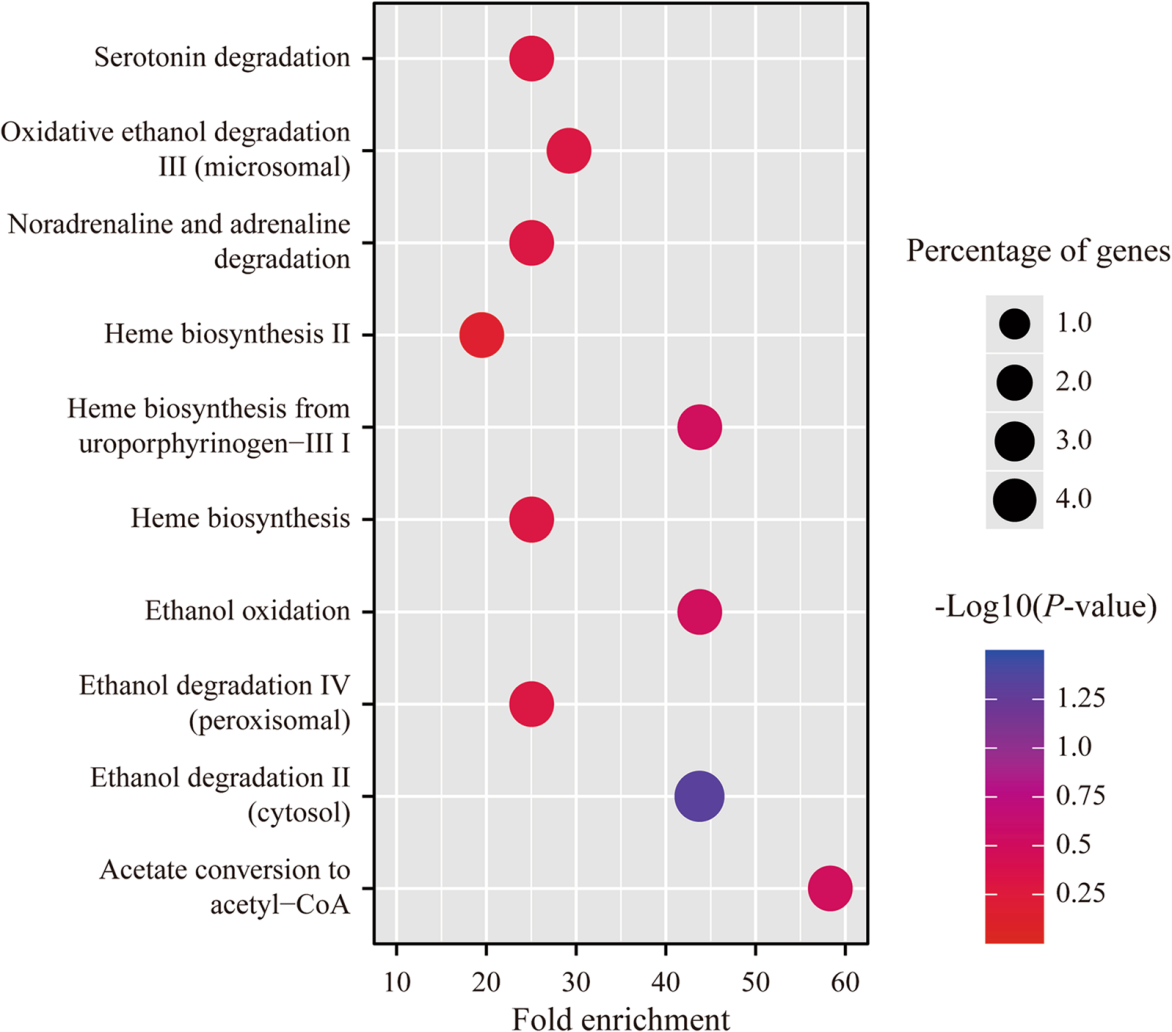
The top 10 significant items in the pathway analysis for the differentially expressed proteins in EBVaGC

Moreover, a pathway analysis was performed to seek the possible biological pathways in which the differentially expressed proteins in EBVaGC might function. The records with top 10 *P* values were also selected. Only one item, ethanol degradation II (cytosol), demonstrated significant enrichment effect for those proteins (*P* = 0.047, fold enrichment = 43.75). And its percentage of enriched genes was 4.2% (Fig. 5).

### Verification for the differentially expressed proteins in EBVaGC with GEO datasets

**Fig. 6:**
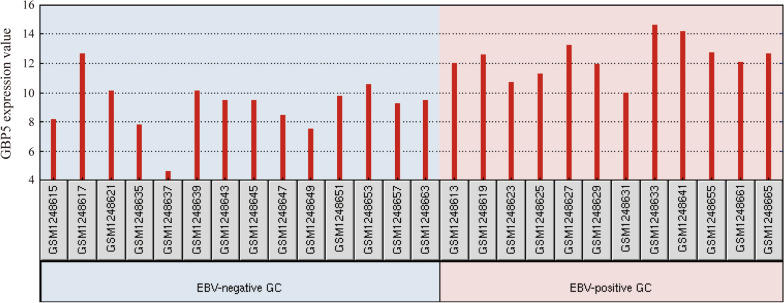
The expression levels of GBP5 gene (mRNA) in EBVaGC from the microarray gene expression profiling (GSE51575) in GEO datasets

To elucidate the features of protein profiles in EBVaGC comprehensively, GEO database was also utilized to search high-throughput experimental data related to EBVaGC. A dataset of microarray gene expression profiling (GSE51575) was retrieved, containing 12 EBV-positive and 14 negative GC cases. We screened all the overlapping genes from differential records between GEO dataset and our array, including 15 up-regulated and 10 down-regulated genes. Interestingly, GBP5 was the only top gene with the highest fold change in both datasets. It was also suggested to be significantly up-regulated in EBV-positive GC compared with EBV-negative GC (*P* = 1.19E-03, log_2_FC = 3.21, Additional file 1: Table S3), indicating that GBP5 might be a highly associated protein with EBVaGC. The expression levels of GBP5 in all tissue samples were presented in Fig. 6.

### Validation for GBP5 expression in EBVaGC

**Fig. 7:**
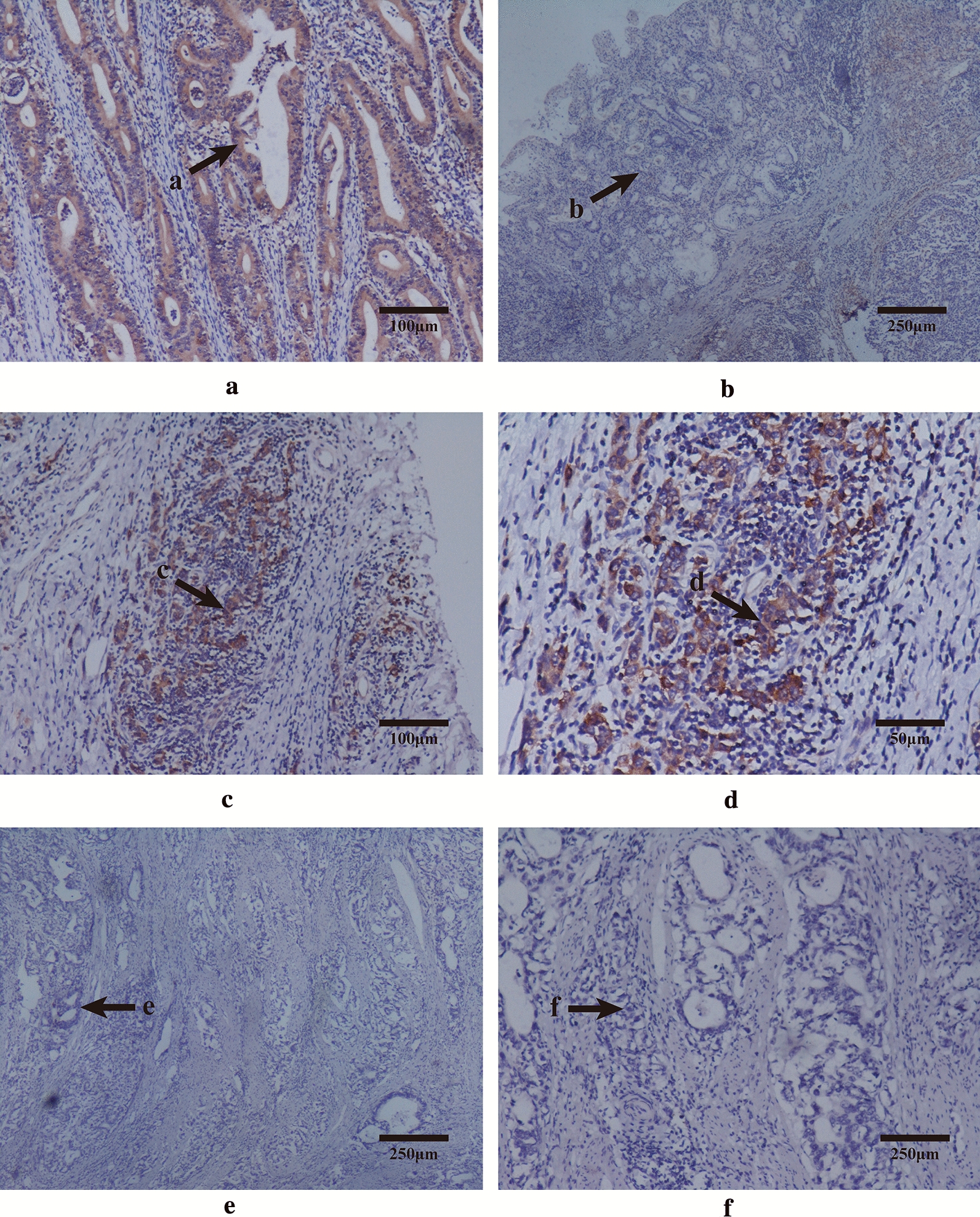
The expression levels of GBP5 protein in EBVaGC by IHC staining. **A**, **a** EBV-positive GC tissue (× 100), positive staining in epithelial cell membrane and cytoplasm (score = 4); **B**, **b** adjacent normal tissue of **A**, **a** (× 40), negative staining in epithelial cell membrane and cytoplasm; **C**, **c** EBV-positive GC tissue (× 100), positive staining in the membrane of lymphocytes (score = 4); D&d, amplified visual field of **C**, **c** (× 200); **E**, **e** EBV-negative GC tissue (× 40), negative staining in epithelial cell membrane and cytoplasm; **F**, **f** EBV-negative GC tissue (× 40), negative staining in the membrane of lymphocytes

Finally, a validation experiment was conducted to confirm the close association of GBP5 protein with EBVaGC. IHC staining was performed to detect GBP5 expression in a total of 255 tissue specimens including 7 EBV-positive and 248 EBV-negative GC cases with their corresponding adjacent normal tissue. The basic characteristics of GC subjects were presented in Additional file 1: Table S4. Representative photomicrographs of tissue cell staining were shown in Fig. 7. In EBV-positive GC, the staining signals of GBP5 protein were brown in color and located in epithelial cell membrane and cytoplasm, while no marked staining was found in adjacent normal tissue (Fig. 7A vs. B). Furthermore, GBP5 protein was also brown-stained in the membrane of lymphocytes among EBV-positive GC tissue (Fig. 7C, D). As for EBV-negative GC, neither epithelium nor mesenchyme has obviously positive staining in tissue specimens (Fig. 7E, F).

Based on the IHC staining results, related analyses for the association of GBP5 protein with GC clinicopathological parameters and prognosis were further performed. Foremost, we found that GBP5 expression had significant or borderline association with multiple GC clinicopathological parameters (Table 2). The positive rates were significantly higher in the following GC subgroups compared with control subgroups, including deeper invasion of gastric wall (muscularis + serosa, *P* = 0.042), positive vascular cancer embolus (*P* = 0.021) and positive extranodal tumor implantation (*P* = 0.011). However, no significant association between GBP5 expression and GC prognosis was found in either univariate or multivariate analysis after adjustment by the impacted factors of overall survival (Additional file 1: Table S5 and Additional file 1: Table S6). Moreover, an additional correlation was observed between GBP5 expression and EBV infection. GBP5 protein tended to be expressed in EBV-positive GC (*P* = 0.054), and its IHC staining score in the 7 EBV-positive GC cases was markedly higher than EBV-negative GC (3.2 ± 1.6 vs. 1.2 ± 1.5, *P* = 0.002, Table 3).

**Table 2:** The association between GBP5 protein expression and clinicopathological parameters of GC

Parameters	GBP5 expression		*P*
	Positive (%)	Negative (%)	
Lauren classification			0.067
Diffuse type	73 (90.1)	109 (80.7)	
Intestinal type	8 (9.9)	26 (19.3)	
Histological type			0.057
Low/un-differentiated	75 (90.4)	109 (80.7)	
High/middle-differentiated	8 (9.6)	26 (19.3)	
Depth of invasion			**0.042**
Muscularis + Serosa	73 (86.9)	102 (75.6)	
Mucosa + Submucosa	11 (13.1)	33 (24.4)	
Growth mode			0.264
Diffuse/invasive	63 (75.0)	109 (81.3)	
Nest	21 (25.0)	25 (18.7)	
Lymphatic metastasis			0.882
Positive	52 (63.4)	83 (62.4)	
Negative	30 (36.6)	50 (37.6)	
Peritumor lymphocyte infiltration			1.000
Positive	82 (98.8)	130 (97.7)	
Negative	1 (1.2)	3 (2.3)	
Vascular cancer embolus			**0.021**
Positive	53 (63.1)	63 (47.0)	
Negative	31 (36.9)	71 (53.0)	
Perineural invasion			0.334
Positive	66 (78.6)	96 (72.7)	
Negative	18 (21.4)	36 (27.3)	
Extranodal tumor implantation			**0.011**
Positive	11 (13.3)	5 (3.8)	
Negative	72 (86.7)	125 (96.2)	

*GC* gastric cancer

The results are in bold if *P* < 0.05

**Table 3:** The association between GBP5 protein expression and EBV infection in GC

Variables	GBP5 expression		
	Positive (%)	Negative (%)	Score
EBV ( +)	6 (10.2)	1 (1.6)	3.2 ± 1.6
EBV (−)	53 (89.8)	63 (98.4)	1.2 ± 1.5
	*P* = 0.054		***P* = 0.002**

The results are in bold if *P* < 0.05

*GC* gastric cancer

## Discussion

Undoubtedly, thorough study for the molecular features of EBVaGC is of great pathological and clinical values. Here, a comprehensive analysis was presented for the protein profile in EBVaGC tissue based on DIA-MS. A total of 137 differentially expressed proteins were identified between EBV-positive and negative GC. PPI network and gene enrichment analysis were successively performed for all differential proteins. Combined with the gene expression profiling in GEO datasets, a highly associated protein (GBP5) with EBVaGC was screened out and validated with IHC staining. As far as we concerned, for the first time our study integrally revealed the protein expression patterns in EBVaGC along with the potential biological function of differentially expressed proteins. In addition, we also firstly reported the highly associated protein with EBVaGC followed by preliminary validation.

Virus-host interactions within infected cells are the core parts during EBV-induced carcinogenesis. Compared with the relatively simple proteomics in virus, the number of genes and complexity of proteomics in host are much more than the former. Besides, the expression levels of various oncogenes and tumor suppressor genes in the infected host cells could vary with the stimulation of viral gene products [15, 16]. Therefore, the proteomic analysis in EBVaGC is quite valuable, and the proteins with remarkable differences and central roles maybe potential diagnostic markers of EBVaGC. Lots of differentially expressed proteins in EBVaGC were newly identified in our study. Although the evidence about their direct relations with EBVaGC is limited, some hints have been manifested in their respective association with EBV infection and GC initiation such as several top proteins like GBP5, C5AR1 and THRAP3 [17–19]. Furthermore, a few crucial genes in EBVaGC were excavated from the differential proteins by means of network analysis. The PPI network showed several proteins with relatively strong interactions such as ITGB2, CDC5L, CYBB and HLA-DRB1. Consistently, previous reports have also suggested that they may serve as hub genes in many diseases especially carcinoma [20–23]. Considering both the differential profile and PPI network, a highly studied hub gene (HLA-DRB1) is noteworthy, which was concurrently one of the top 10 up-regulated records in the assay. Its expression and polymorphisms were shown to be associated with both EBV infection and GC [24, 25]. In general, the establishment of protein profiles in EBVaGC greatly improved the access to its molecular research. The key proteins with significantly differential expression and hub roles could be selected as potential biomarkers for EBVaGC detection. However, substantial discovery studies are needed to confirm that.

The specific programs of viral gene expression found in EBVaGC can target cell signaling pathways leading to increased proliferation, cell survival, immune invasion, augmented epithelial-to-mesenchymal transition (EMT) and acquisition of stemness features [15]. For instance, Zhao et al. reported 13 pathways deregulated in EBVaGC, including mitogen-activated protein kinase (MAPK), Wnt and focal adhesion etc., which could facilitate rapid tumor growth [26, 27]. Coincidently, some differential proteins mentioned above were indicated to participate in the genesis of gastric adenocarcinoma or stromal tumors via these classical pathways such as GBP5, C5AR1 and THRAP3 [28–30]. Beyond that, EBVaGC-specific cellular pathways have also been increasingly explored [11]. For example, Want et al. found alterations in macromolecular biosynthetic processes, and deregulation of cholesterol transport and lipoprotein clearance pathways was also evident in EBVaGC [26, 31]. Novel findings were observed in our prediction for the biological function of differentially expressed proteins in EBVaGC. They were shown to be enriched in the metabolic pathways of energy including mitochondrion or biochemical substances like ethanol degradation, along with catalytic activity. The metabolic landscape of EBVaGC was investigated before and aberrant metabolism in EBVaGC was well accepted. Significant down-regulation of genes involved in metabolic pathways has been proved especially biochemical metabolism such as amino acids, lipids and carbohydrates [32, 33]. So far, however, rare study has referred to the change of energy pathways in EBVaGC. Only one gene set enrichment analysis by Sohn et al. revealed that EBVaGC had significant genetic alterations in pathways involving energy production [34]. Some clues could be extracted from the association between EBVaGC and mitochondrion-related pathways. An original research showed that EBV-encoded BARF1 was down-regulated in EBV-positive malignant cells and induced caspase-dependent apoptosis via mitochondrial pathway [35]. Another report suggested that the expression of CCL21 by EBVaGC cells protected CD8( +) CCR7( +) T lymphocytes from apoptosis via mitochondria-mediated pathway [36]. Therefore, it is reasonable to infer that the differential proteins in EBVaGC might function in the dysregulation of energy metabolism by mediating mitochondrial pathways, and even affect the survival of EBV-infected GC cells. Nevertheless, all the hypotheses about concrete mechanisms need further verification.

Combined our high-throughput assay with public database, a highly associated protein of EBVaGC, GBP5, was found out with the highest fold change of differential expression both in the present study and GEO dataset. IHC staining also confirmed its overexpression in EBVaGC tissue. GBP5 (Guanylate binding protein 5) is a member of IFN-inducible subfamily of guanosine triphosphatases (GTPases) and exert critical roles in cell-intrinsic immunity against diverse pathogens including EBV [37]. The expression level of GBP5 was increased in the peripheral blood mononuclear cells of patients with chronic active EBV infection [18]. The involvement of GBP5 in the immune microenvironment of GC has also been preliminarily explored. A previous IHC experiment demonstrated that GBP5 had cytoplasmic and membranous expression in GC cells while no signals in non-neoplastic stomach [30]. Meanwhile, EBV could invade into B-lymphocytes, epithelial cells and fibroblasts through different mechanisms, thus the up-regulation of GBP5 might appear in both epithelia and mesenchyme. All these phenomena were consistent with our assay. Moreover, further analyses revealed that GBP5 protein was correlated with some malignant GC clinicopathological features. Considering GBP5 also took parts in innate immune activation and the regulation of inflammasomes related to cancer [38], its overexpression might be defensively activated in lesion when poor differentiation arose in GC cells. Importantly, GBP5 protein was validated to have a higher expression trend in GC tissue with EBV infection compared with EBV-negative GC, which laid a more convinced association with EBVaGC. Hence, GBP5 protein could be a promising EBVaGC-related marker with the function as an anti-EBV factor and effector of immune defense against GC progression simultaneously, in spite of the need to further investigation.

To be acknowledged, however, only the most representative protein GBP5 was validated with IHC and further analyzed. More proteins with the potential to be EBVaGC-related markers except for GBP5 might be hidden in other differential records from DIA-MS or GEO database. And it is quite necessary to validate them in future studies.

## Conclusions

In summary, we conducted a comprehensive analysis of the protein profile in EBVaGC mainly by the aid of DIA-MS. A few differentially expressed proteins were newly identified between EBV-positive and negative GC, and several hub genes were subsequently revealed. The proteins with significant differences and potential central roles could be applied as diagnostic markers of EBVaGC. They were also predicted to be involved in the biological pathways related to energy and biochemical metabolism. Additionally, a highly associated protein (GBP5) was screened out by a joint analysis with GEO database and validated with IHC staining, which might be a key protein in EBVaGC. Our study could provide research clues for EBVaGC pathogenesis as well as novel targets for the molecular-targeted therapy of EBVaGC.

## Supplementary Information

**Additional file 1:** **Figure S1.** The EBER-ISH staining of 7 EBV-positive GC cases (A1-A7). Positive signals are brown-stained.

**Additional file 2:** **Table S1.** The basic characteristics of GC cases to be assayed. **Table S2.** The raw quantity of differentially expressed proteins in GC samples. **Table S3.** The overlapping differential genes between DIA-MS and GEO datasets. **Table S4.** The basic characteristics of GC subjects for GBP5 validation. **Table S5.** The association between host characteristics and overall survival of GC patients. **Table S6.** The association between GBP5 protein expression and GC prognosis.

## Data Availability

All data generated of analyzed during this study are included in this published article.

## Abbreviations

EBV: Epstein-Barr virus
EBVaGC: Epstein-Barr virus-associated gastric cancer
TCGA: The Cancer Genome Atlas
HE: Hematoxylin–eosin
EBER: Epstein-Barr virus-encoded RNA
ISH: In situ hybridization
DIA: Data-independent acquisition
MS: Mass spectrometry
FASP: Filter-aided sample preparation
HPLC: High performance liquid chromatography
DDA: Data-dependent acquisition
IHC: Immunohistochemistry
HRP: Horseradish peroxidase
FC: Fold change
FDR: False discovery rate
PPI: Protein–protein interaction
GO: Gene Ontology
GEO: Gene Expression Omnibus
BH: Benjamini-Hochberg
CRC: Colorectal cancer
CC: Cellular component
MF: Molecular function
BP: Biological process
EMT: Epithelial-to-mesenchymal transition
MAPK: Mitogen-activated protein kinase
GBP5: Guanylate binding protein 5
GTPases: Guanosine triphosphatases
