# Supplementary material for: Identification of differential proteomics in Epstein-Barr virus-associated gastric cancer and related functional analysis
Source: Cancer Cell Int. 2021 Jul 12;21:368. doi: 10.1186/s12935-021-02077-6 (PMC8274036; doi:10.1186/s12935-021-02077-6)
Supplement: Supplementary file 1 — Additional file 1: Figure S1. The EBER-ISH staining of 7 EBV-positive GC cases (A1-A7). Positive signals are brown-stained. [file 12935_2021_2077_MOESM1_ESM.docx]

| Table S1.The basic characteristics of GC cases to be assayed | | | | | | |
| --- | --- | --- | --- | --- | --- | --- |
| Samples | Gender | Age | Tumor cite | Macroscopic type | Histological type | Lymphatic metastasis |
| A1 | male | 49 | corpus | protruded type | middle differentiation | positive |
| A2 | male | 62 | corpus | ulcerative type | low differentiation | positive |
| A3 | male | 65 | gastric stump | ulcerative infiltrative type | low differentiation | negative |
| A4 | male | 73 | whole stomach | diffuse infiltrative type | low differentiation | positive |
| A5 | male | 83 | corpus and angle | ulcerative infiltrative type | low differentiation | positive |
| A6 | male | 49 | antrum | ulcerative type | lymphoepitehlioma-like | negative |
| A7 | male | 59 | corpus and angle | ulcerative type | low differentiation | positive |
| B1 | male | 49 | corpus and angle | diffuse infiltrative type | low differentiation | positive |
| B2 | male | 65 | corpus | diffuse infiltrative type | low differentiation | positive |
| B3 | male | 68 | corpus | ulcerative infiltrative type | middle/low differentiation | negative |
| B4 | male | 69 | whole stomach | diffuse infiltrative type | low differentiation | positive |
| B5 | male | 83 | angle | ulcerative infiltrative type | low differentiation | positive |
| B6 | male | 52 | antrum | ulcerative infiltrative type | low differentiation | negative |
| B7 | male | 63 | corpus and angle | diffuse infiltrative type | low differentiation | positive |
| Note: GC, gastric cancer. | | | | | | |

| Table S2. The raw quantity of differentially expressed proteins in GC samples | | | | | | | | | | | | | | |
| --- | --- | --- | --- | --- | --- | --- | --- | --- | --- | --- | --- | --- | --- | --- |
| Genes | A1 | A2 | A3 | A4 | A5 | A6 | A7 | B1 | B2 | B3 | B4 | B5 | B6 | B7 |
| GBP5 | 306954.3 | 14865.1 | 236390.4 | 96098.0 | 144909.5 | 216627.3 | 229229.0 | 26031.0 | 7487.0 | 145266.6 | 20322.3 | 58735.5 |  |  |
| C5AR1 | 165204.9 | 633225.1 | 471937.6 |  | 142286.8 | 146327.0 | 356771.8 | 98128.6 |  |  | 89551.1 | 114483.8 | 47686.7 | 121146.4 |
| THRAP3 | 266163.1 | 324922.3 | 259618.7 |  |  | 196924.1 |  |  |  | 86582.9 | 26807.6 |  | 85116.5 | 123575.5 |
| P3H3 | 38705.2 | 150363.2 | 45268.1 |  | 111096.4 |  | 87472.7 |  | 22274.3 |  | 24368.0 | 23416.8 | 3620.8 | 65973.9 |
| MDK | 111243.5 | 113430.4 | 184347.2 |  |  | 82053.6 | 40581.8 | 6286.1 |  | 34110.8 |  | 30553.1 | 67617.8 |  |
| ALOX5AP | 1208335.5 | 5847291.0 | 2791722.5 | 542987.4 | 1514326.8 | 2198522.8 | 1795087.5 | 614542.0 | 1180654.8 | 1398837.5 | 817932.8 | 458561.4 | 429287.5 | 705804.5 |
| BPI | 207093.8 | 396187.7 | 144719.8 |  | 473967.8 | 68503.3 | 442717.1 | 76416.4 | 150101.4 | 94787.6 | 230174.6 | 51141.1 | 16112.9 | 131704.3 |
| HLA-DRB1 | 877525.6 |  |  |  | 870628.6 | 710624.2 | 364010.9 |  | 285752.6 | 485043.3 | 377908.3 |  | 81309.3 | 150655.6 |
| PPL | 34217.8 | 131816.3 |  | 53460.0 | 117258.3 |  | 95527.4 | 33659.8 | 45794.1 | 11471.0 |  | 12328.3 | 71069.8 | 34134.6 |
| ISLR |  | 495893.6 | 241192.4 |  | 253232.8 | 66081.7 | 468738.1 | 80736.7 | 166393.4 |  | 211084.0 | 133775.4 | 95158.1 | 104770.7 |
| APOL2 | 259628.5 | 101494.4 | 257245.2 | 155839.7 | 269505.4 | 234111.0 | 387735.8 | 111822.7 | 56340.5 | 145261.1 |  | 180146.5 | 56125.2 | 74209.6 |
| HCK | 39532.9 | 104070.0 | 72600.3 |  | 53061.4 | 45506.4 | 77501.9 | 36999.8 |  | 64424.0 | 31926.9 | 13876.7 | 6359.7 | 24117.9 |
| AKAP2 | 68497.7 | 122887.7 |  | 88520.1 | 45056.2 |  | 69514.9 | 26846.3 |  |  | 45223.8 | 40350.4 | 33338.1 |  |
| ITGA11 |  | 56172.9 | 37558.7 |  | 31268.7 |  | 42885.1 | 20112.6 |  |  | 19367.8 | 32349.1 | 6496.1 |  |
| ITGB2 | 571055.3 | 1313069.6 | 615259.1 | 361760.5 | 400015.0 | 528640.2 | 773049.9 | 303338.5 | 314051.8 | 593220.4 | 231016.5 | 258674.2 | 95434.4 | 348235.6 |
| COQ6 | 406258.3 | 770343.8 | 397193.2 | 387378.5 | 345383.3 | 847463.9 |  | 239593.8 | 215406.5 | 684638.8 | 236885.5 | 133446.2 | 82992.6 | 171383.6 |
| DENND1C | 59391.5 | 34476.4 | 56312.3 | 42324.6 | 47480.8 | 63802.2 |  | 17576.6 |  | 29938.0 | 36676.6 | 22302.2 |  | 16350.9 |
| RAB31 | 37903.5 | 111917.9 | 72181.4 | 23452.3 | 78852.9 | 32675.5 | 38364.7 | 26490.9 | 41636.9 | 33061.7 | 23241.8 | 34496.9 | 11925.9 | 21595.2 |
| CYBA | 957543.6 | 1585900.0 | 1359879.8 | 593390.8 | 941893.9 | 1253949.9 | 1153309.5 | 459120.8 | 641808.1 | 639526.6 | 665512.3 | 728802.5 | 337990.9 | 413168.3 |
| FCGR3A |  |  | 495428.4 | 288540.6 | 335330.9 | 187342.3 | 691979.3 | 164115.7 | 138744.2 | 279267.4 | 243626.2 | 199560.4 |  | 207973.4 |
| CYBB | 378420.2 | 859214.9 | 690973.5 | 325728.6 | 392531.4 | 505328.8 | 621202.4 | 346611.6 | 289590.2 | 396808.7 | 288493.9 | 256166.5 | 170181.4 | 239260.3 |
| KEAP1 | 105049.5 | 76595.0 |  | 75309.1 |  |  | 85381.8 |  |  | 31799.3 | 41526.5 | 51886.8 | 36183.9 | 66248.5 |
| KALRN | 156562.9 | 142712.9 | 202872.6 | 162014.7 | 156907.1 | 150057.8 |  | 32622.8 | 69734.5 | 130923.3 | 95978.1 | 92667.9 | 101182.4 | 87093.9 |
| GBP1 | 660819.4 | 129347.5 | 711298.8 | 507704.4 | 606777.8 | 559301.1 | 740814.1 | 242056.6 | 226653.6 | 419694.2 | 480520.4 | 455833.0 | 136663.4 | 151349.0 |
| DPYD | 59780.4 | 50638.2 | 119940.1 | 62500.2 | 105215.4 | 76134.6 | 180580.2 | 57432.5 | 86320.1 | 51231.5 | 68203.7 | 25642.1 | 42525.3 | 30210.8 |
| TOR1B | 39945.1 | 55532.7 | 69628.3 | 49616.8 | 37197.9 | 38489.3 | 55825.5 | 27332.2 |  |  |  | 40897.3 | 14896.4 | 26710.3 |
| CNN2 | 302582.3 | 694407.4 | 266731.1 | 434298.4 | 242841.9 | 386992.4 | 766445.9 | 256346.4 | 283786.5 | 278401.9 | 381230.0 | 151577.7 | 224121.0 | 164455.8 |
| TCIRG1 | 168481.2 | 293962.3 | 220377.9 | 81106.2 | 151142.6 | 141949.1 | 136855.8 | 86175.4 |  | 103933.6 | 106310.2 | 95515.4 | 99064.2 | 84430.6 |
| TAP1 | 1691408.8 | 513617.8 | 1948572.5 | 1612332.5 | 2836836.3 | 2130154.5 | 1285552.8 | 775619.9 | 534123.2 | 1585759.8 | 1093768.6 | 1470100.1 | 578362.9 | 780916.8 |
| SRRM2 | 116113.6 | 86081.4 | 84082.7 |  |  | 90078.1 |  |  |  | 69824.8 |  | 61870.6 | 63537.8 | 19254.8 |
| CD40 | 433842.3 |  | 189359.8 | 300488.2 | 384653.8 | 213248.1 | 240627.2 | 166966.9 | 127200.6 | 321960.9 |  | 158217.1 | 86204.5 | 150096.7 |
| FUT8 | 148014.7 | 117597.5 | 247803.7 | 140288.9 | 248567.8 | 157356.1 | 370797.7 | 178372.1 | 128048.1 | 112409.0 | 85049.1 | 82228.9 | 120248.5 | 131451.5 |
| SCAF1 | 11557.4 |  | 23225.7 |  |  | 24952.7 | 22887.7 | 4370.1 |  | 11287.2 | 11387.7 | 11968.0 | 14115.2 | 19997.7 |
| TLR3 | 28039.7 |  | 72088.2 | 39171.4 | 58808.2 | 64192.8 | 47608.2 | 46647.3 | 26069.4 | 37831.5 | 28412.0 | 20750.9 | 39519.1 | 18856.3 |
| GRN | 132035.4 | 249436.2 | 209081.1 | 139067.6 | 170359.5 | 138021.5 | 307729.2 | 68732.2 | 194928.1 | 65627.2 | 117034.0 | 147583.9 | 95946.3 | 124340.0 |
| NSA2 | 29698.1 | 71171.4 | 76116.6 | 53757.7 |  | 51473.3 | 41098.5 | 57560.1 | 28283.3 | 34441.2 | 16317.3 | 19733.3 |  | 40272.7 |
| CLASP1 | 109319.0 | 181776.2 | 129996.3 | 82017.4 | 95777.6 | 110801.1 | 192720.6 | 130186.2 | 93497.7 | 34439.2 | 54461.7 | 98885.8 | 56633.5 | 91937.9 |
| CPOX | 116472.9 | 157132.3 | 116421.6 | 77753.8 | 91617.3 | 120663.3 | 198673.3 | 91531.5 | 90450.3 | 52797.1 | 78838.0 | 61369.3 | 49623.8 | 121257.2 |
| ATP6AP1 | 158054.4 | 298375.5 | 268010.5 | 194484.5 | 211080.0 | 180569.1 | 281900.0 | 52074.4 | 216760.3 | 170930.4 | 172422.2 | 167710.0 | 170323.5 | 45404.5 |
| CARHSP1 | 159425.3 | 151409.1 | 137687.0 | 41598.3 | 84024.5 | 149073.8 | 154558.7 | 97884.3 | 87437.6 | 51637.0 | 83429.4 | 60362.7 | 123743.8 | 44479.4 |
| LPCAT2 | 127708.6 | 224736.5 | 219080.5 | 91685.7 | 99974.3 | 167610.5 | 196905.6 | 134827.2 | 59406.3 | 89116.8 | 60809.4 | 93462.4 | 97682.7 | 173193.5 |
| GALNT2 | 212087.8 | 242452.7 | 429513.5 | 205033.4 | 288769.4 | 246747.0 | 518203.2 | 244362.6 | 185067.4 | 249002.5 | 115436.4 | 173033.9 | 196088.2 | 187834.5 |
| COMMD10 | 118904.7 | 91572.7 |  |  | 128821.7 | 84789.9 |  | 87014.3 |  |  | 78546.8 | 82035.2 | 51043.6 | 35763.4 |
| ATP6V1D | 111150.3 | 125945.0 | 222961.1 | 115808.0 | 101906.9 | 135377.9 | 190937.2 | 84498.9 | 101169.9 | 116362.6 | 86261.2 | 98919.8 | 43181.3 | 107335.2 |
| LRRC40 | 89873.4 | 130086.7 | 100000.3 | 56017.7 | 87855.9 | 78888.7 | 85028.7 |  | 55116.7 | 51593.6 | 49374.2 | 48175.5 | 81605.0 | 63101.6 |
| PREX1 | 53992.1 | 58264.9 | 76974.9 | 33250.5 | 60649.7 | 75886.5 | 46501.5 | 47460.0 | 42934.3 | 63575.3 | 29549.5 | 30876.1 | 18629.0 | 31746.4 |
| GBP2 | 295815.3 | 91447.0 | 288925.5 | 203993.4 | 217845.5 | 256349.2 | 210463.0 | 181332.7 | 120498.3 | 188934.6 | 143794.9 | 181818.7 | 100212.0 | 107031.4 |
| PEBP1 | 2432016.8 | 1522965.5 | 2023978.3 | 3149811.3 | 2254376.8 | 2414097.0 | 1358065.6 | 3037261.0 | 4972360.0 | 3337995.5 | 2709261.8 | 2377195.5 | 3483337.8 | 2898872.8 |
| UBR5 | 17279.1 | 14825.9 | 21130.1 | 27741.0 |  | 14554.8 | 14933.1 | 16068.4 | 30009.7 | 34000.0 | 25477.7 | 20858.4 |  | 40532.7 |
| TXN2 | 54179.4 | 37064.9 | 54785.1 | 61310.4 | 35807.0 | 52322.4 | 55097.7 | 61813.4 | 76190.7 | 76901.9 | 92021.7 | 61708.4 | 111885.1 | 51087.7 |
| ADD1 | 369732.3 | 156091.2 | 288297.3 | 447374.4 | 305115.6 | 353430.7 | 251477.3 | 394158.4 | 719623.6 | 542329.6 | 474765.0 | 386360.7 | 414613.6 | 362318.4 |
| EPB41L1 | 436446.7 | 377385.7 | 140714.1 | 333697.5 | 299860.3 | 225666.8 | 155340.7 | 448226.0 | 459360.3 | 524075.9 | 288146.3 | 283837.3 | 396191.4 | 602462.6 |
| IDI1 | 51595.0 | 79686.0 | 50961.5 | 59263.9 | 54221.2 | 38058.7 | 41547.2 | 73521.8 | 93414.3 | 79990.0 | 96716.9 | 47236.6 | 76046.3 | 111587.5 |
| EML2 | 166244.1 | 101742.3 | 131153.4 | 153198.3 | 117966.9 | 155121.6 | 113434.9 | 223918.3 | 219694.2 | 157568.7 | 131012.5 | 174863.2 | 369970.6 | 179412.6 |
| ATP1B1 | 755464.8 | 482416.1 | 321650.8 | 825820.5 | 328870.3 | 661847.4 | 376790.9 | 1053911.6 | 1299223.9 | 611766.8 | 676368.3 | 763252.9 | 815998.3 | 614986.8 |
| EIF4A2 | 356966.6 | 245008.0 | 342176.2 | 378094.6 | 517673.4 | 392892.0 | 439175.6 | 479338.7 | 864824.8 | 467004.5 | 515934.3 | 599602.9 | 647528.8 | 592580.1 |
| MRI1 | 17255.6 | 51617.2 | 31744.6 | 25245.2 | 39864.8 | 24867.2 | 33606.7 | 51474.0 | 58943.8 | 40101.8 | 44447.7 | 42187.5 | 44672.2 | 68250.7 |
| CST3 | 390927.5 | 314749.0 | 310974.1 | 409798.7 | 290661.9 | 312279.8 | 255722.8 | 438327.8 |  | 266474.5 | 441768.6 | 448371.5 | 829268.6 | 638327.6 |
| ABHD14B | 285705.5 | 260780.0 | 187479.9 | 229828.6 | 231550.8 | 228709.4 | 271770.3 | 361456.9 | 434665.7 | 292348.0 | 493976.0 | 431372.9 | 490572.9 | 157120.2 |
| ARFIP2 | 56653.4 | 72614.7 | 52331.8 | 44351.6 | 50062.2 | 37605.1 | 40516.7 | 69424.5 |  |  |  | 51785.3 | 102250.6 | 95389.3 |
| ATPAF2 | 70573.7 | 56485.3 | 22415.7 | 68625.2 | 41259.0 | 70364.3 | 51014.9 | 94731.7 | 55472.3 | 72661.6 | 61074.3 | 96305.9 | 110735.0 | 110007.2 |
| PSMG4 | 41652.8 |  |  | 22977.1 | 34478.2 | 20436.3 | 22280.0 | 43144.6 |  |  |  | 53421.5 | 31599.5 | 52276.0 |
| ECSIT | 12009.4 | 24622.8 | 10457.0 | 34665.8 | 20588.2 | 14431.7 | 10183.8 | 34896.2 | 38561.4 | 20803.9 | 19962.8 | 32785.0 | 29975.7 | 25174.5 |
| RNMT | 54212.6 | 62694.8 | 43938.3 | 36899.9 | 42678.3 | 49310.4 | 23926.6 | 92529.8 | 84475.4 | 48288.4 | 83555.4 | 41583.5 | 53668.1 | 95974.3 |
| CD46 | 156239.4 | 147769.2 | 123676.8 | 137179.6 | 190736.7 | 90906.1 | 118953.8 | 189244.7 | 271065.9 | 157726.8 | 217038.8 | 231208.9 | 359992.8 | 124984.8 |
| SUPV3L1 | 36141.1 | 82092.0 | 58278.8 | 99936.4 | 69838.0 | 60641.5 | 63724.7 | 124378.6 | 179810.8 | 97830.1 | 41440.6 | 82426.9 | 101471.1 | 130094.3 |
| DTD1 | 306041.6 | 282815.8 | 244958.2 | 288916.5 | 267146.2 | 447849.2 |  | 332243.2 | 714298.3 | 451159.8 | 597913.1 | 516703.2 | 454616.2 | 388239.1 |
| FAM213A | 117409.3 | 143857.4 | 143794.8 | 82256.1 | 116861.1 | 91258.1 | 119305.8 | 222759.3 | 299463.6 | 181120.3 | 96641.7 | 144662.2 | 148309.2 | 235482.2 |
| C11orf54 | 194140.7 | 150814.2 | 152494.7 | 333499.2 | 183417.4 | 128592.6 | 77346.8 | 389833.9 | 452049.2 | 295939.6 | 210991.4 | 200899.3 | 282602.9 | 159082.3 |
| BCKDHB | 94333.5 | 91544.2 | 79560.5 | 155238.5 | 166470.8 | 121297.9 | 53598.0 | 198598.7 | 195868.4 | 234810.0 | 150402.6 | 100030.1 | 219375.6 | 151386.5 |
| GFPT1 | 962961.5 | 544986.5 | 366598.7 | 744078.7 | 625845.3 | 480777.8 | 484593.5 | 1342033.1 | 751046.8 | 767197.4 | 657327.9 | 872792.7 | 1755824.0 | 772508.1 |
| EPB41L2 | 245492.2 | 424575.4 | 771947.2 | 171634.8 | 692595.5 | 460661.8 | 481864.4 | 707873.2 | 701037.6 | 435136.8 | 1245719.0 | 820680.3 | 820685.9 | 609084.8 |
| RAB6D/RAB6C | 87284.5 | 80095.5 | 67340.9 | 58094.9 | 96852.1 | 73350.7 | 70504.0 | 101029.8 | 151586.6 | 101924.2 | 73525.7 | 114868.9 | 114234.0 | 226193.2 |
| DAG1 | 95275.1 | 69758.3 | 106717.2 | 91986.3 | 93178.9 | 66632.6 |  | 164976.6 | 206330.1 | 82087.1 | 177700.4 | 117881.3 | 178982.5 | 88944.5 |
| HEBP2 | 218047.1 | 162973.4 | 164699.5 | 184721.1 | 117929.7 | 143197.4 | 184892.6 | 388858.0 | 438428.3 | 165290.8 | 132934.1 | 210465.6 | 394162.3 | 231730.3 |
| QDPR | 57389.7 | 44071.4 | 69721.1 | 84284.5 | 58458.3 | 49431.4 | 42623.3 | 138888.9 | 135142.2 | 29151.5 | 127440.0 | 70401.4 | 82599.8 |  |
| UBE4B |  | 13551.0 | 17229.8 | 7755.2 | 26390.5 |  |  | 30374.2 |  | 26471.6 | 19706.2 | 24575.2 |  | 35177.1 |
| NAXE | 124920.5 | 166222.2 | 267540.8 | 213034.0 | 177396.7 | 150702.6 | 156251.8 | 316800.9 | 429305.2 | 211707.3 | 285178.0 | 195602.5 | 397395.2 | 273981.2 |
| GLRX5 | 64715.0 | 98409.1 | 80814.5 | 31955.4 | 86720.7 | 70352.0 | 85708.6 | 164343.5 | 159581.6 | 119682.7 | 59085.9 | 124661.3 | 127707.4 | 124549.0 |
| PPOX | 32721.4 |  | 45949.3 | 77676.5 | 61708.3 | 67291.6 | 25387.1 | 52032.0 | 94423.5 | 95102.8 |  | 78387.3 | 97888.1 | 109951.8 |
| CHRAC1 | 31165.0 | 27933.2 | 24244.3 | 19932.8 | 16390.5 | 27499.5 | 14260.3 |  | 31642.6 |  | 53834.4 | 58032.1 | 13444.8 | 40077.4 |
| MPST | 192283.6 | 192592.1 | 178041.3 | 306723.9 | 196369.2 | 122334.8 | 161762.6 | 213420.8 | 321949.1 | 184606.3 | 339438.4 | 427671.9 | 529331.3 | 321541.0 |
| COQ3 | 24692.9 | 53266.4 | 35654.0 | 51112.9 | 59182.6 | 33909.4 | 25700.8 | 76132.7 | 103939.3 | 54443.6 | 49367.5 | 45105.6 | 61881.7 | 100368.0 |
| F13A1 | 122877.5 | 231827.7 | 112392.5 | 423466.0 | 475493.4 | 166612.1 | 220604.8 | 481258.4 | 299854.1 | 461814.7 | 405813.5 | 215954.6 | 537538.2 | 644433.9 |
| SGCD | 48224.9 | 129975.5 | 63541.4 | 67632.9 | 102907.3 | 45752.5 | 109814.2 | 176616.1 | 184246.7 |  | 178389.8 | 138307.7 | 102001.4 | 70011.8 |
| NFU1 | 45092.4 | 85285.9 | 90446.8 | 73558.5 | 48310.8 | 44568.1 | 48727.8 | 128755.6 | 97719.8 | 37795.2 | 97509.7 | 137654.6 | 161489.7 | 103514.7 |
| TXLNG | 25634.1 | 33809.6 | 11343.1 | 15228.0 | 16225.3 | 10517.1 | 27543.1 | 38431.8 |  | 32317.7 | 34720.7 | 26458.6 |  | 44538.7 |
| NRM | 18618.6 |  | 41258.4 | 74233.1 | 89806.9 | 29278.3 | 24137.6 | 96400.8 | 108701.1 | 62042.3 | 77661.2 | 70675.7 | 77485.1 |  |
| ACAA2 | 772642.6 | 459659.3 | 671322.0 | 548267.5 | 1262545.4 | 1180675.1 | 632874.5 | 905160.2 | 1453907.4 | 1191825.5 | 1080258.1 | 1492265.1 | 2413599.5 | 1317956.3 |
| TXNL4A | 27353.5 | 21036.0 | 23666.7 | 48454.9 | 23787.6 | 29742.4 | 32756.5 | 45121.5 | 58726.2 | 62311.0 | 89819.8 | 27664.6 | 26092.5 | 63160.5 |
| F11R | 153943.8 | 288424.3 | 136658.6 | 185009.7 | 157887.4 | 148195.1 | 209058.8 | 379975.6 | 521592.8 | 317391.2 | 206081.1 | 186460.0 | 311350.5 | 384773.6 |
| H2AFY2 | 123565.4 | 142601.9 | 198269.1 | 132999.1 | 222122.2 | 190848.4 |  | 229645.8 | 280552.6 | 359198.0 | 159641.7 | 259338.8 | 318999.6 | 523828.9 |
| SPRYD4 | 124667.3 | 143887.9 | 66527.9 | 147596.8 | 82055.8 | 78929.9 | 65183.4 | 162999.9 | 91699.8 | 231318.0 | 101525.3 | 100744.9 | 305438.8 | 296288.1 |
| RIDA | 68596.6 | 134370.7 | 154741.8 | 90006.0 | 62024.6 | 87339.4 | 119580.5 | 145127.4 | 215677.3 | 235637.6 | 153364.5 | 253026.3 | 243229.3 | 66931.0 |
| MLYCD | 21319.8 | 18228.0 | 32061.6 | 25732.0 | 35730.8 | 23638.1 |  | 55697.7 | 51901.1 |  | 32091.8 | 37040.8 | 71774.5 | 41419.3 |
| ACY1 | 59963.2 | 124812.5 | 60883.2 | 79825.8 |  | 44115.7 | 91022.1 | 145877.3 | 185688.5 | 131977.9 | 120355.7 | 151998.0 | 135947.5 | 133315.9 |
| CDC5L | 150884.4 | 154452.2 | 149423.8 | 293689.6 | 90374.3 | 299208.2 | 127172.8 | 258053.0 | 518140.3 | 307691.4 | 505996.6 | 325264.2 | 298307.8 | 163636.8 |
| ACSS2 | 132788.9 | 75250.2 | 22862.3 | 120338.4 | 53117.9 | 76406.4 | 52231.4 | 166540.1 | 204097.6 | 84672.0 | 78935.3 | 125246.7 | 190669.3 | 158960.2 |
| DARS2 | 113371.8 | 117393.5 | 100905.1 | 197543.2 | 156445.8 | 105242.2 | 110979.4 | 162650.3 | 303186.5 | 259096.8 | 162917.6 | 190407.3 | 459860.7 | 210753.0 |
| 2-Mar | 60641.6 |  | 102532.5 | 122401.3 |  | 75483.7 |  | 170340.3 | 210291.5 | 158831.3 |  | 101556.7 | 220852.5 | 198550.0 |
| CA1 | 780216.3 | 1425517.6 | 499467.5 | 979475.0 | 1352665.5 | 1196899.5 | 312360.8 | 587743.9 | 1719057.0 | 1749900.9 | 2852090.8 | 1649312.3 | 1388118.0 | 3084710.0 |
| BRK1 | 66075.0 | 46535.9 | 69953.4 | 117482.6 | 88001.9 | 37396.8 | 52144.2 | 141155.7 | 147396.3 | 99819.5 | 82802.4 | 202363.5 | 144986.9 |  |
| CAVIN2 | 44419.7 | 41726.0 | 42590.3 | 35410.8 | 43376.5 | 42287.3 | 19457.4 | 142721.9 | 110699.3 | 35440.3 | 68914.4 | 66280.7 | 92745.8 | 27785.0 |
| SELENBP1 | 1006037.3 | 415971.4 | 628989.0 | 1257592.5 | 552143.1 | 394771.1 | 354918.2 | 1977144.3 | 1590413.8 | 467843.0 | 1192912.6 | 797564.1 | 2389659.8 | 937587.8 |
| COQ8A | 55376.7 | 31022.5 | 59855.7 | 97492.3 | 66863.7 | 47687.3 |  | 70326.7 | 117974.2 | 139121.5 | 69351.0 |  | 219814.5 | 112735.6 |
| HBG1 | 30868272.0 | 56815832.0 | 20700902.0 | 25274148.0 | 34118588.0 | 47591492.0 | 2245807.0 | 37905400.0 | 30819746.0 | 58383584.0 | 105335144.0 | 95249840.0 | 53103108.0 | 69271704.0 |
| PFN2 | 210980.8 | 475410.7 | 299007.7 | 226258.8 | 417157.6 | 303943.9 | 263593.1 | 671065.3 | 777316.8 | 505372.2 | 810252.4 | 513556.6 | 557186.7 | 719132.9 |
| ARHGEF10 | 4983.2 | 6114.5 |  | 6248.9 | 5837.1 |  |  | 11272.1 | 13460.6 |  | 8005.2 | 15577.7 | 12089.8 |  |
| GRIP2 | 42598.8 | 51558.9 | 142287.9 | 158495.5 | 122143.5 | 171691.6 |  | 124015.0 | 199233.5 | 462316.8 | 217787.9 | 246707.0 | 269996.9 | 184982.2 |
| SH3BGRL2 | 110813.6 | 111848.4 | 57221.1 | 150293.3 | 15810.7 | 33085.0 |  | 221263.7 | 325760.3 | 139873.7 | 124152.8 | 148627.4 | 120126.0 | 114308.0 |
| TMEM63A | 74729.2 | 66872.7 | 73507.6 | 111396.2 | 48953.1 | 112232.4 |  | 104748.6 |  |  |  | 201104.9 | 305687.9 | 95629.4 |
| CRAT | 28022.4 | 15876.2 | 52802.2 | 26816.5 | 49569.3 | 42173.8 | 31814.9 | 66769.5 | 99490.8 | 74171.3 | 94636.0 | 40458.6 | 113701.0 | 50350.0 |
| HBE1 | 23351528.0 | 38256224.0 | 14256828.0 | 18100210.0 | 28298414.0 | 37469140.0 | 2818861.3 | 23411024.0 | 23503658.0 | 40134720.0 | 79651840.0 | 50118388.0 | 44650800.0 | 105342472.0 |
| IGKV2-24 |  | 133823.3 | 72864.5 | 176577.7 | 94471.5 | 164818.4 | 184346.0 | 163924.9 | 456270.8 | 404683.7 | 370126.9 | 161103.6 | 506329.4 | 135338.2 |
| VWA5A | 81229.6 | 37299.7 | 59767.7 | 129333.1 | 121213.0 | 108871.8 | 50268.5 | 219215.3 | 360904.4 | 118411.8 | 234329.0 | 143479.8 | 236802.8 | 74396.3 |
| MAOB |  |  | 268724.7 | 99452.6 | 64350.1 | 305668.4 |  | 384894.6 | 539561.0 | 398062.9 | 361998.8 | 342053.6 | 671527.5 | 369318.4 |
| DEPTOR | 30613.9 |  | 32471.7 | 34681.8 |  | 21326.4 |  | 93554.9 |  | 36659.9 | 60213.3 | 94435.3 | 71154.3 |  |
| LTBP4 | 235971.4 | 47934.5 | 255000.6 | 369947.8 | 65250.9 | 352774.2 |  | 520013.6 | 1075027.4 | 227763.7 | 453859.5 | 580651.1 | 531312.0 | 318953.5 |
| THADA | 13595.7 |  |  | 7693.2 |  | 4895.2 | 8290.9 | 19565.4 | 23215.6 | 20278.8 |  | 13938.1 | 10031.2 | 39408.8 |
| ACSS1 | 102529.5 | 10911.5 | 70737.9 | 126973.5 | 103240.5 | 143328.0 | 25554.8 | 284908.3 | 329272.4 | 150883.8 | 60024.6 | 147885.5 | 338714.7 | 115721.4 |
| ASS1 | 234518.1 |  |  | 55936.1 | 249563.9 | 53018.4 |  | 482607.5 | 300034.3 |  | 473759.9 | 234446.4 | 412402.3 | 289563.0 |
| EPHB3 | 38813.7 | 20870.0 | 30214.7 | 69151.0 | 24252.1 | 18062.8 | 24250.9 | 50333.7 |  | 38974.1 | 147905.9 | 92000.9 | 53933.3 | 107339.6 |
| ADH1B | 1030392.8 | 143820.0 | 589132.6 | 1829701.6 | 285951.3 | 1104673.8 | 171491.2 | 3009470.5 | 2121259.3 | 548435.7 | 2112617.3 | 654296.3 | 4040506.5 | 1143571.8 |
| HMGCS1 | 54918.5 |  | 25818.3 | 23764.2 |  | 34686.5 | 61225.6 | 219248.2 | 41612.5 | 121096.5 |  | 71638.7 | 95637.3 | 88041.0 |
| SLC12A2 | 109881.6 | 38263.8 | 85691.9 | 147297.7 | 44332.9 | 83056.1 |  | 336188.8 | 219469.2 | 95842.3 | 228040.2 | 291870.5 | 284287.3 | 158609.7 |
| PTGR1 | 27551.6 | 39940.5 | 94590.8 | 101888.2 | 83721.9 | 67316.3 | 33252.7 | 204929.2 | 110375.7 | 136381.8 | 216185.7 | 104130.5 | 299488.1 | 152069.3 |
| PHGDH | 174308.1 | 123923.3 | 91462.9 | 162793.0 | 349226.1 | 204948.8 | 90815.3 | 684781.1 | 238088.1 | 723450.6 | 191184.1 | 440309.7 | 399807.5 | 610796.3 |
| LRRC1 | 40279.5 | 38423.3 | 35358.6 | 8117.1 | 6842.5 | 19551.3 | 9612.6 | 93347.5 |  | 42579.1 |  | 23538.8 | 68499.4 | 82810.4 |
| FAF1 | 32789.7 | 15971.3 | 17238.5 | 15734.0 | 17893.0 | 16369.0 |  | 100603.5 |  | 29458.8 | 68776.0 | 44208.1 | 69982.4 | 18626.4 |
| OPLAH | 98414.3 | 33861.4 | 32803.5 | 125329.1 | 34454.3 | 46647.5 | 87245.9 | 105217.0 | 289849.7 | 79149.3 | 177962.5 | 262753.8 | 229416.2 | 170308.0 |
| CKMT1A | 635858.1 | 369005.1 | 111551.5 | 588262.4 | 110508.6 | 209002.9 | 220374.3 | 926155.6 | 2002941.5 | 265064.3 | 254650.0 | 1097289.0 | 1654877.3 | 323120.9 |
| CEP250 | 170870.9 | 154796.2 | 149850.9 | 515857.0 | 175002.1 | 158266.6 |  |  | 840949.4 |  | 509041.4 | 484178.0 | 980176.4 |  |
| BCAM |  | 74782.9 | 73788.5 | 209737.9 | 199355.7 | 93908.5 | 72010.6 | 140697.5 | 378360.4 | 702559.1 | 921422.6 | 235815.2 | 162472.4 | 451702.2 |
| EPHB2 |  |  | 15609.6 | 18704.9 |  | 16483.4 | 23316.2 |  | 94119.3 |  | 115949.7 | 42215.3 |  | 29082.4 |
| MFAP4 | 101344.3 | 204863.8 | 268218.6 | 198801.2 | 49493.7 | 297418.1 | 73004.7 | 676908.6 | 1457999.5 | 228621.4 | 1503078.4 | 612523.9 | 336700.5 | 62123.0 |
| AKR7A3 | 323842.5 | 56637.7 | 140590.4 | 284561.0 | 66327.2 | 45586.9 | 57615.8 | 841752.4 | 1303633.8 | 103671.8 |  | 154587.1 | 657453.6 | 372524.5 |
| TMEM168 | 2407.4 | 12649.1 | 10561.2 | 3989.4 | 3288.0 | 3225.3 |  | 43823.2 | 6947.5 |  |  | 33771.5 | 25359.6 |  |
| Note: GC, gastric cancer. | | | | | | | | | | | | | | |

| Table S3. The overlapping differential genes between DIA-MS and GEO datasets | | | | | |
| --- | --- | --- | --- | --- | --- |
|  |  |  |  |  |  |
| Genes | FC (abs) | *P* value | Regulation | GEO-*P* | GEO-Log_2_FC |
| GBP5 | 3.45 | 0.028 | Up | 0.001 | 3.21 |
| ALOX5AP | 2.84 | 0.048 | Up | 0.015 | 1.49 |
| HLA-DRB1 | 2.56 | 0.015 | Up | 0.001 | 2.78 |
| APOL2 | 2.29 | 0.009 | Up | 0.020 | 1.69 |
| HCK | 2.21 | 0.020 | Up | 0.011 | 1.39 |
| ITGB2 | 2.13 | 0.025 | Up | 0.005 | 2.10 |
| FCGR3A | 1.94 | 0.044 | Up | 0.036 | 1.87 |
| CYBB | 1.90 | 0.007 | Up | 0.011 | 2.12 |
| GBP1 | 1.85 | 0.020 | Up | 0.012 | 2.06 |
| DPYD | 1.81 | 0.049 | Up | 0.007 | 1.66 |
| TAP1 | 1.76 | 0.037 | Up | 0.007 | 1.71 |
| CD40 | 1.74 | 0.036 | Up | 0.010 | 1.86 |
| FUT8 | 1.71 | 0.037 | Up | 0.044 | 1.04 |
| PREX1 | 1.53 | 0.029 | Up | 0.010 | 1.49 |
| GBP2 | 1.53 | 0.023 | Up | 0.004 | 1.64 |
| NRM | 1.78 | 0.026 | Down | 0.023 | -1.04 |
| ACAA2 | 1.78 | 0.015 | Down | 0.035 | -1.12 |
| MLYCD | 1.85 | 0.007 | Down | 0.011 | -1.09 |
| ACY1 | 1.87 | 0.001 | Down | 0.004 | -1.51 |
| DARS2 | 1.94 | 0.014 | Down | 0.002 | -1.36 |
| PFN2 | 2.07 | <0.001 | Down | 0.015 | -2.07 |
| ASS1 | 2.47 | 0.013 | Down | 0.045 | -2.39 |
| HMGCS1 | 2.65 | 0.046 | Down | 0.005 | -1.33 |
| PHGDH | 2.75 | 0.005 | Down | 0.010 | -2.79 |
| BCAM | 3.55 | 0.029 | Down | 0.004 | -1.16 |
| Note: DIA-MS, Data-independent acquisition mass spectrometry; GEO, Gene Expression Omnibus; FC (abs), absolute fold change. | | | | | |
|  |  |  |  |  |  |

| Table S4. The basic characteristics of GC subjects for GBP5 validation | | |
| --- | --- | --- |
|  |  |  |
| Information | GBP5 expression | |
|  | Positive (%) | Negative (%) |
| Total | n=98 | n=157 |
| Gender | *P*=0.252 | |
| Male | 48 (75.0) | 70 (66.7) |
| Female | 16 (25.0) | 35 (33.3) |
| Age | *P*=0.842 | |
| Mean±SD | 60.7±11.2 | 60.5±10.0 |
| Median | 60 | 62.5 |
| Range | 25-85 | 26-80 |
| *H.pylori* infection | *P*=0.117 | |
| Positive | 56 (76.7) | 82 (66.1) |
| Negative | 17 (23.3) | 42 (33.9) |
| Smoking | *P*=0.593 | |
| Ever | 15 (28.3) | 22 (32.8) |
| Never | 38 (71.7) | 45 (67.2) |
| Drinking | *P*=0.195 | |
| Ever | 15 (27.8) | 12 (17.9) |
| Never | 39 (72.2) | 55 (82.1) |
| Note: GC, gastric cancer. | | |

| Table S5. The association between host characteristics and overall survival of GC patients | | | | |
| --- | --- | --- | --- | --- |
|  |  |  |  |  |
| Factors | GC patients | Death | MST (m) | *P* |
| Total | n=189 | n=76 |  |  |
| Gender |  |  |  | 0.673 |
| Male | 104 | 36 | 50.5^a^ |  |
| Female | 42 | 12 | 73.1 |  |
| Age |  |  |  | 0.936 |
| ≥60y | 112 | 44 | 73.1 |  |
| <60y | 77 | 32 | 49.4^a^ |  |
| *H.pylori* infection |  |  |  | 0.989 |
| Positive | 106 | 43 | 73.1 |  |
| Negative | 49 | 19 | 49.5^a^ |  |
| Smoking |  |  |  | 0.303 |
| Ever | 33 | 15 | 38.0 |  |
| Never | 66 | 34 | 73.1 |  |
| Drinking |  |  |  | 0.237 |
| Ever | 23 | 12 | 38.0 |  |
| Never | 77 | 38 | 73.1 |  |
| Lauren classification |  |  |  | 0.097 |
| Diffuse type | 157 | 69 | 73.1 |  |
| Intestinal type | 30 | 6 | 60.7^a^ |  |
| Histological type |  |  |  | 0.097 |
| Low/un-differentiated | 158 | 69 | 73.1 |  |
| High/middle-differentiated | 30 | 6 | 60.7^a^ |  |
| Depth of invasion |  |  |  | **<0.001** |
| Muscularis+Serosa | 152 | 72 | NA |  |
| Mucosa+Submucosa | 37 | 4 | NA |  |
| Growth mode |  |  |  | **0.001** |
| Diffuse/invasive | 147 | 69 | 38.0 |  |
| Nest | 41 | 7 | 67.9^a^ |  |
| Lymphatic metastasis |  |  |  | **<0.001** |
| Positive | 117 | 65 | 30.0 |  |
| Negative | 68 | 9 | 73.1 |  |
| Peritumor lymphocyte infiltration | |  |  | 0.927 |
| Positive | 183 | 72 | 49.6^a^ |  |
| Negative | 4 | 3 | 73.1 |  |
| Vascular cancer embolus |  |  |  | **0.018** |
| Positive | 102 | 51 | 42.0 |  |
| Negative | 86 | 25 | 57.2^a^ |  |
| Perineural invasion |  |  |  | **<0.001** |
| Positive | 142 | 69 | 36.0 |  |
| Negative | 44 | 6 | 70.3^a^ |  |
| Extranodal tumor implantation |  |  |  | **<0.001** |
| Positive | 16 | 13 | 8.0 |  |
| Negative | 168 | 61 | 73.1 |  |
| Note: GC, gastric cancer; MST (m), median survival time (month); ^a^, mean survival time was adopted when MST could not be calculated. The results are in bold if *P*<0.05. | | | | |
|  |  |  |  |  |

| Table S6. The association between GBP5 protein expression and GC prognosis | | | |
| --- | --- | --- | --- |
|  |  |  |  |
| Variables | MST (m) | *P* | HR (95%CI) |
| Univariate |  |  |  |
| GBP5 (+) | 73.1 | 0.925 | 1.03 (0.54-1.97) |
| GBP5 (-) | 49.7^a^ |  | 1 (Ref) |
| Multivariate |  |  |  |
| GBP5 (+) | 73.1 | 0.460 | 1.30 (0.65-2.60) |
| GBP5 (-) | 49.7^a^ |  | 1 (Ref) |
| Note: GC, gastric cancer; MST (m), median survival time (month); ^a^, mean survival time was adopted when MST could not be calculated; HR, hazard ratio; CI, confidence interval. | | | |
|  |  |  |  |
|  |  |  |  |
