# Supplementary figures and images for: Identification of differential proteomics in Epstein-Barr virus-associated gastric cancer and related functional analysis
Source: Cancer Cell Int. 2021 Jul 12;21:368. doi: 10.1186/s12935-021-02077-6 (PMC8274036; doi:10.1186/s12935-021-02077-6)

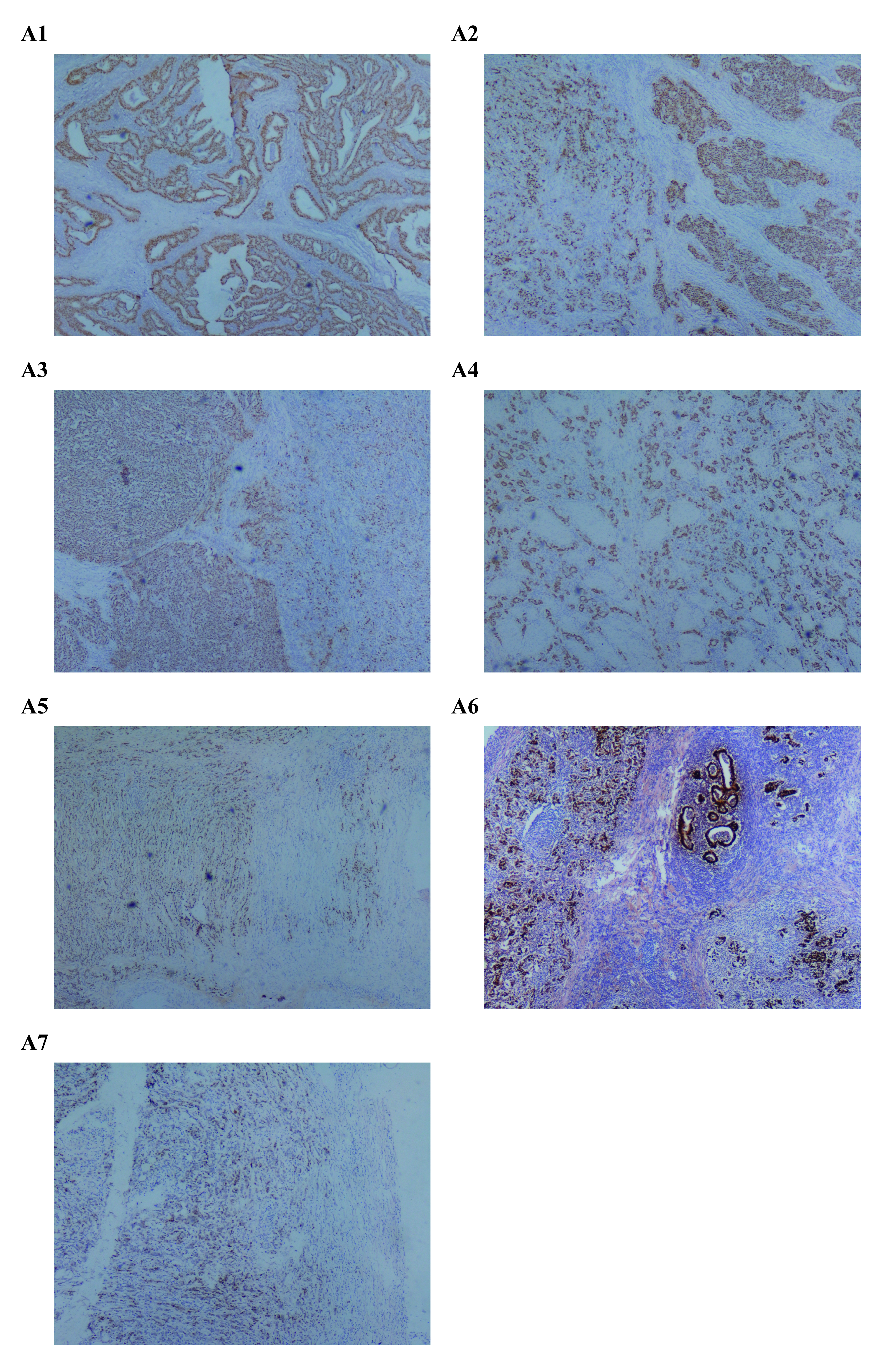

Supplement: Supplementary file 2 — Additional file 2: Table S1. The basic characteristics of GC cases to be assayed. Table S2. The raw quantity of differentially expressed proteins in GC samples. Table S3. The overlapping differential genes between DIA-MS and GEO datasets. Table S4. The basic characteristics of GC subjects for GBP5 validation. Table S5. The association between host characteristics and overall survival of GC patients. Table S6. The association between GBP5 protein expression and GC prognosis. [file 12935_2021_2077_MOESM2_ESM.tif]
